# MicroRNA-mediated susceptible poplar gene expression regulation associated with the infection of virulent *Melampsora larici-populina*

**DOI:** 10.1186/s12864-015-2286-6

**Published:** 2016-01-15

**Authors:** Danlei Li, Feng Wang, Chao Wang, Li Zou, Zhiying Wang, Qiaoli Chen, Chunyang Niu, Ruizhi Zhang, Yaming Ling, Bowen Wang

**Affiliations:** College of Forestry, Northeast Forestry University, Harbin, 150040 China

**Keywords:** *Melampsora larici-populina*, *Populus*, Susceptibility, microRNAs, Degradome, DGE

## Abstract

**Background:**

Rust caused by *Melampsora larici-populina* is one of the most damaging diseases of poplars. Rust is considered to be a model pathogen for genetic studies because both pathogen and host genomes are available. The poplar ‘Robusta’, whose general rust resistance is defeated by virulent rust E4, provides suitable host material for studies of the gene regulation involved in rust resistance/susceptibility. In this study, we investigated the microRNA-mediated susceptible poplar gene expression regulation associated with the infection of virulent rust. We were particularly interested in delineating the host-pathogen interactions with a specific focus on microRNAs (miRNAs).

**Results:**

To study the susceptibility of poplar to *M. larici-populina*, small RNA (sRNA) libraries, a degradome cDNA library and digital gene expression libraries were constructed for rust-inoculated and rust-free susceptible poplar ‘Robusta’ leaves through high-throughput sequencing. Altogether, 12,722 regulating interactions were identified. The results delineated the framework of post-transcriptional regulation of gene expression in the susceptible poplar, which was infected by the virulent rust. The results indicated that pathogen-associated molecular patterns (PAMPs) and PAMP-triggered immunity were induced by the infection of virulent rust E4 and that miRNAs still functioned at this stage. After this stage, miRNA-regulated *R* genes, such as TIR-NBS-LRR and CC-NBS-LRR, were not fully functional. Additionally, the rust-responsive miRNAs did not regulate the signaling component genes related to the salicylic acid pathway or the hypersensitive response.

**Conclusions:**

We found that the defense-related post-transcriptional regulation of the susceptible poplar ‘Robusta’ functions normally only at the stage of PAMPs and PAMP-triggered immunity (PTI). More importantly, the miRNA-mediated post-transcriptional regulation of defense signal pathway genes were inactivated by the infection of virulent rust at the stage of effector-triggered susceptibility and during the following stages of salicylic acid and hypersensitive responses. This inactivation was the major characteristic of ‘Robusta’ susceptibility.

**Electronic supplementary material:**

The online version of this article (doi:10.1186/s12864-015-2286-6) contains supplementary material, which is available to authorized users.

## Background

Rust caused by *Melampsora larici-populina* is one of the most severe diseases of poplars. Rust is indigenous to Eurasia and later spread to Australia [1] and North America [2]. In the Far East, *M. larici-populina* infection occurs in a wide range of poplar species and hybrids and often causes severe damages [3, 4]. It has a very complex life cycle, producing 5 spore stages during its life cycle and alternating on larch (*Larix* spp.).

Poplars (*Populus* spp.) occur naturally in most parts of the northern hemisphere from subarctic to subtropical regions [5] and are widely planted beyond their natural range in both the southern and northern hemispheres [6]. Poplars and their hybrids vary in rust resistance. Although hybrids of *P. deltoides* and *P. nigra* or *P. trichocarpa* were selected for their immunity to rust [7], breakdown of the resistance to *M. larici-populina* in some of these cultivars was detected upon the appearance of races E1 and E2 [8, 9]. Subsequently, many cultivars that had been highly resistant to rust were infected by a new race, E4 [10]. Resistance and susceptibility are at opposite ends of the same spectrum, and research on disease resistance cannot be conducted without reference to susceptibility [11]. Although great advances have been made in researching the genetic mechanisms of plant disease resistance, the mechanisms underlying plant disease susceptibility remain unclear.

*Populus deltoides* possesses qualitative resistance to *M. larici-populina* [12]. Although hybrid vigor is generally high in these cultivars, most of the hybrids exhibit high rust susceptibility once the major resistance genes inherited from *P. deltoides* are defeated [12, 13]. In a previous study, hybrid poplar *P. nigra* × *P. deltoides* ‘Robusta’ was found to have non-race-specific resistance to *M. larici-populina* isolates collected from England [4], but the resistance was defeated by E4. Therefore, ‘Robusta’ provides suitable host material for studies of gene regulation involved in rust resistance/susceptibility.

MicroRNAs (miRNAs) play important roles in plant post-transcriptional gene regulation by repressing the translation of target mRNAs or targeting them for cleavage [14]. In plants, miRNAs regulate diverse processes, such as development [12], abiotic stress tolerance [15], and biotic stress [16]. Several studies have indicated that miRNAs play critical roles in disease resistance or susceptibility responses [16]. For example, several plant miRNAs, such as miR160, miR167, miR398 and miR1885, are responsive to biotic stress during both resistance and susceptibility interactions [17–19]. Additionally, in susceptible poplar, all fungi-responsive miRNAs have been found to be up-regulated in response to the infection of a canker pathogen [20].

The genome sequences of both poplar (*P. trichocarpa*) and its rust pathogen *M. larici-populina* have now been published [21]. Thus, the poplar–poplar rust pathogen system is the only system in which the genome sequences of both tree host and rust pathogen are available. Although poplar genomic analyses have revealed the genetic potential of susceptibility and transcriptomics has allowed for the deciphering of gene expression in space and time, little is known about the post-transcriptional regulation of resistance gene expression. Recent evidence has suggested that post-transcriptional silencing involving small RNAs of cellular rather than pathogenic origin might have broad implications in potentiating basal defense and race-specific resistance to microbes in plants [22]. The present study was conducted to decipher the regulatory functions of miRNA in a susceptible poplar and to unravel the complexity of post-transcriptional regulation of resistance genes in the susceptible poplar ‘Robusta’.

## Results

### Small RNA sequencing profile

To study the post-transcriptional regulation associated with poplar susceptibility to rust, miRNA accumulation was investigated by small RNA sequencing. Two small RNA (sRNA) libraries constructed from a combination of seven selected time points of the ‘Robusta’ leaves of -rust (rust-free) and + rust (E4 rust-inoculated) were sequenced on an Illumina GAIIx system. To acquire more details of the sRNA background in one sequencing, seven time points covering the major developmental transitions of the urediniospores in poplar leaves were selected, consistently with previous studies [23, 24]. A total of 8,840,983 (Additional file 1: Figure S1 A) and 9,736,262 (Additional file 1: Figure S1 B) raw sequences were generated from the -rust and + rust libraries, respectively. Blast homology searches were performed against *M. larici-populina* (version 1.0, JGI website http://genome.jgi-psf.org/Mellp1/), and the mapped reads were removed from the + rust library.

The length distribution of the mappable reads showed that 15-nt reads (18 %) were the most abundant sequences in the + rust sRNA library and that 21-nt reads (17.5 %) were the second most abundant sequences (Additional file 1: Figure S2). In contrast, the -rust sRNA library showed that 21-nt reads were the most abundant sequences (Additional file 1: Figure S2), constituting 19.3 % of all reads. This latter result is in agreement with results from sRNA studies of *P. balsamifera* [25] and *Vitis vinifera* [26], in which the 21-nt sRNAs showed the highest abundance. However, this result differs from the results of studies of other plants, in which 24-nt sRNAs were most abundant [27–28].

By removing the redundant sequences from mappable reads, 3,820 and 4,070 unique sequences were identified from the -rust and + rust libraries, respectively. After removing the consensus sequences, a total of 5,138 unique miRNAs were identified in these two sRNA libraries. These unique miRNAs were classified into two groups: known miRNAs and predicted miRNAs (Additional file 1: Figure S1 C and D, Additional file 1: Table S1). Known miRNAs were further divided into two subgroups: Gp1a and Gp1b (Additional file 1: Text S1). Predicted miRNAs were further divided into six subgroups: Gp2a, Gp2b, Gp3a, Gp3b, Gp4a and Gp4b (Additional file 1: Text S1). By removing the no-hairpin subgroups and the subgroups that did not map to the poplar genome, Gp1a, Gp1b, Gp2a and Gp4a were identified as candidate miRNA subgroups. Among these candidate miRNA subgroups, 1,474 and 1,475 miRNAs were obtained from the -rust and + rust sRNA libraries, respectively. The potential novel miRNAs identified by deep sequencing were deposited in The European Nucleotide Archive (WEBIN ID number: Hx2000050201).

According to the eleven features of the miRNA hairpins (LC Sciences, Houston, TX, USA, Additional file 1: Text S2), the secondary structure of genes corresponding to the potential novel miRNAs (Gp4a) were identified (Table 1), and 4 predicted secondary structures of the potential novel miRNAs are shown as examples (Additional file 1: Figure S3).

**Table 1:** The potential novel miRNAs and their targets

miRNA			Targets	
miRNA	log_2_	Sequences (5′-3′)	Annomination	log_2_
PC-5p-310376_3	12.64	UUUUCAAUAAUUGCAUCAAUA	NB-ARC domain-containing disease resistance protein	−0.37
PC-3p-1638036_1	11.06	UUUGAUAGAACCACUGCA	xyloglucan endotransglucosylase/hydrolase 28	0.38
PC-5p-674947_1	11.06	GCAGUGACUUGAAAGAA	RING-box 1	−0.00
PC-3p-2607690_1	2.77	CGGCAACGGAAUUUAUUUUAUU	E3 ubiquitin ligase SCF complex subunit SKP1/ASK1 family protein	0.03
PC-5p-236891_4	2.27	UCUCAACGAAACUUCAAUCGU	Sec23/s protein transport family protein	−0.23
PC-3p-142486_6	1.77	UUACACAGAACCAUGCC	Golgi nucleotide sugar transporter 4	0.17
PC-3p-2198351_1	1.18	ACUCGUGAUUUUAACAACCUUGGU	RING/FYVE/PHD-type zinc finger family protein	−0.14
PC-3p-390998_2	1.18	CUGGCAGGGAUUGUAACUGUG	HXXXD-type acyl-transferase family protein	0.14
PC-3p-1014196_1	1.18	CAAAGAAGCUGCAAUUUGAAA	LRR and NB-ARC domains-containing disease resistance protein	−0.54
PC-5p-86003_12	1.18	CUUCUCCAUGGAGUGCAUG	Ferritin 2	−0.49
PC-3p-193248_4	1.03	CCUGGACACUGUUCACU	Polyketide cyclase/dehydrase and lipid transport superfamily protein	0.08
PC-5p-275646_3	−1.14	CGAAGAGUUCUUGGUGUUUU	Preprotein translocase SecA family protein	−0.70
PC-5p-336387_2	−1.40	GUAGUUCUUUCCCAUCAAACC	Phosphoglycerate kinase 1	−0.09
PC-3p-123255_8	−1.40	AAAUUGAUGAAUUUAUGGAGU	WRKY DNA-binding protein 3	0.06
PC-3p-401772_2	−1.40	UUGAGAUUGUAGUAGCAGUGAU	Jasmonate-zim-domain protein 1	−1.29
PC-3p-1525453_1	−10.88	CUGCCCUCGAGAGACUC	Multidrug resistance-associated protein 6	0.14
PC-3p-2241919_1	−10.88	UGUGUUGUAGUAAUUACU	Chaperone protein htpG family protein	−1.10
PC-5p-1903277_1	0.18	ATGTTATGTGGTAGATAA	Aldehyde dehydrogenase 3H1	−1.02
PC-3p-1909651_1	−9.88	ACTCATAATGTTGCAAC	Glutathione S-transferase 6	0.54
PC-5p-359200_2	−10.88	TTACTCGATAATGATATGAAC	Cysteine-rich RLK (RECEPTOR-like protein kinase) 25	1.23
PC-5p-2592253_1	−10.88	AGATAAATCCAACGATACCAAAAA	Cysteine-rich RLK (RECEPTOR-like protein kinase) 42	1.02
PC-5p-1977684_1	−9.88	ATTCCACTGATTTCTTTTATGATG	Cysteine-rich RLK (RECEPTOR-like protein kinase) 26	1.12
PC-3p-719252_1	−9.88	GTTTAATTTCACACACACACACAC	Cysteine-rich RLK (RECEPTOR-like protein kinase) 26	1.00
PC-5p-1490392_1	−9.88	TTTGTGAATTCCAAAAGTGGT	Cysteine-rich RLK (RECEPTOR-like protein kinase) 29	1.13
PC-5p-1501811_1	−9.88	ATTCCCTCGATATATACCGATAGA	Cysteine-rich RLK (RECEPTOR-like protein kinase) 34	1.81
PC-3p-2480743_1	0.18	GAAGTGATCAACATAGAAATGATTT	Cysteine-rich RLK (RECEPTOR-like protein kinase) 42	8.64
PC-5p-1349045_1	−9.88	CATTTGTTCATGCTTCTTA	Disease resistance protein (CC-NBS-LRR class) family	−0.08
PC-5p-1150271_1	10.06	TTGAATTAGAGAAATTAGGGT	SID2	−0.19
PC-5p-1312398_1	−10.88	TGCCCCTTGAATGCCATTCTT	OPR3	0.92
PC-3p-272434_3	−1.82	AGACACACCAAAACTTCAATGATG	MPK4	−0.19

### Identification of rust-responsive miRNA in *P. nigra* × *P. deltoides*

**Fig. 1:**
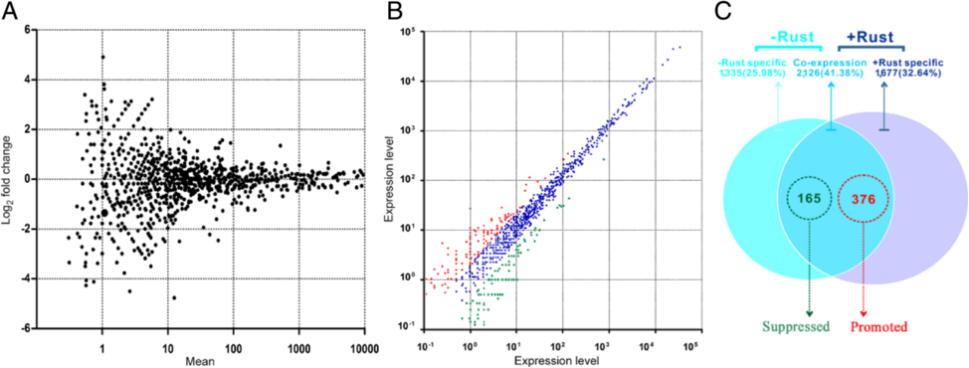
Identification of rust-responsive miRNAs in *P. nigra* × *P. deltoides* ‘Robusta’. **a** Scatter plot of gene expression in + rust and –rust libraries according the log_2_ of normalized reads. Expression levels are normalized by the mean numbers of transcripts per million clean reads (TPM). **b** Scatter plot of miRNA expression levels according to the raw reads. Data points below (green) or above (red) the slope line represent down-regulated or up-regulated miRNAs. **c** Comparison of differentially expressed miRNAs between the -rust and + rust libraries. The Venn diagram displays the distribution of 5,138 unique miRNAs between -rust (left, cyan circle) and + rust (right, slate circle) libraries. The log_2_- (+rust/-rust) fold changes >1 were designated as indicating ‘up-regulated miRNAs’, and the changes < −1 were designated as indicating ‘down-regulated miRNAs’

MiRNAs regulate gene expression in plants by promoting the degradation of or repressing the translation of target mRNAs, both transcriptionally and post-transcriptionally [18, 28]. To associate rust fungi *M. larici-populina* infection with an alteration of poplar miRNA accumulation, the differential expression of miRNAs in the two libraries was compared using the log_2_- (+rust/-rust) fold changes of the normalized miRNA copy numbers (Fig. 1a). Log_2_- (+rust/-rust) fold changes >1 were designated as indicating ‘up-regulated miRNA’ , and changes < −1 were designated as indicating ‘down-regulated miRNA’ (Fig. 1b).

Analysis of the differential expression of 5,138 miRNAs showed that 2,126 (41.38 %) were expressed in both the -rust and + rust libraries (co-expression, Fig. 1c), whereas 1,335 (25.98 %) and 1,677 (32.64 %) were preferentially expressed in -rust and + rust libraries, respectively (Fig. 1c). Analysis of co-expressed miRNAs resulted in the identification of 541 miRNAs (out of 2,126; 25.45 %) that showed significant differential expression between the -rust and + rust libraries. Of these, 376 (73.15 %) were up-regulated, and 165 (32.10 %) were down-regulated after the inoculation of rust.

**Fig. 2:**
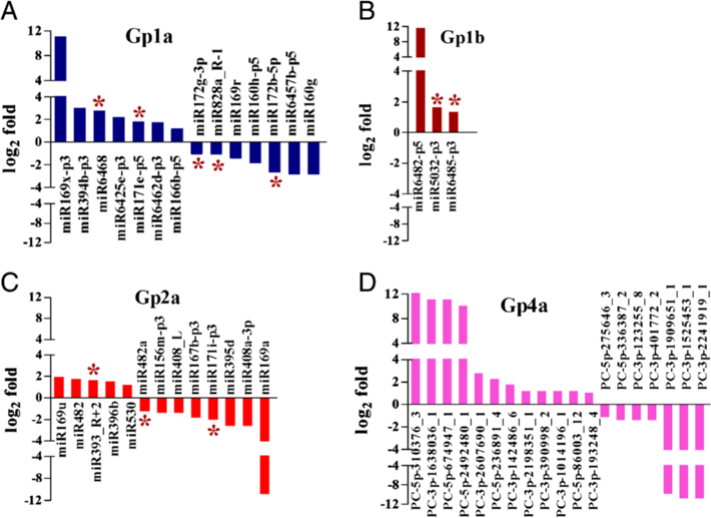
Profiles of the differentially expressed miRNAs. **a** the differentially expressed Gp1a miRNAs;**b** the differentially expressed Gp1b miRNAs; **c** the differentially expressed Gp2a miRNAs; **d** the differentially expressed Gp4a miRNAs. Profiles of the differentially expressed Gp1a, Gp1b, Gp2a and Gp4a miRNAs responsive to rust fungi *M. larici-populina* infection identified in sRNA libraries from infected (+rust) and uninfected (−rust) *P. nigra* × *P. deltoides* ‘Robusta’ plants*.* Only miRNAs with more than 5 raw reads in both of the two libraries are shown. The positive and negative values indicate miRNAs whose expression was up-regulated and down-regulated by rust, respectively. The asterisks denote the expression of the miRNAs at different time points as tested by RT-qPCR

In the four candidate miRNA subgroups, 1,081 miRNA members were responsive to rust infection (log_2_-fold changes >1 or < −1). Restricting the analysis to only those miRNAs with more than 5 raw reads in both of the two sRNA libraries revealed changes of only 61 miRNAs as shown in Fig. 2. In the subgroup Gp1a, 7 miRNAs were up-regulated and 7 miRNAs were down-regulated in response to rust infection (Fig. 2a). With the exception of 3 up-regulated miRNAs, no differentially expressed miRNAs were found in the Gp1b subgroup (Fig. 2b). In the subgroup Gp2a, 5 miRNAs were up-regulated and 8 miRNAs were down-regulated in response to rust infection (Fig. 2c). In total, 12 miRNAs were up-regulated and 7 were down-regulated in response to rust infection in the Gp4a subgroup and were potentially novel miRNAs (Fig. 2d). The most robustly changed miRNA was PC-5p-310376_3 (Gp4a, Fig. 2d), with a peak log_2_-fold change of 12.641.

### Identification of miRNA targets

To identify genes targeted by miRNAs, a degradome cDNA library from + rust poplar leaves was constructed. In total, 8,409,874 raw, short sequencing reads were obtained from this library (Additional file 1: Table S2), and 5,077,704 unique reads mapped to the *Populus* mRNA database (http://www.ncbi.nlm.nih.gov/nuccore/). A list of all of the confidently detected mRNA targets along with the corresponding alignments for the miRNA–mRNA interactions were generated by the pipeline. All five categories (Additional file 1: Text S3) [29] of the sliced-target transcripts were found. According to the 1,475 miRNAs identified in the + rust sRNA library, 947 (64.20 %) miRNAs potentially targeted to 8,463 poplar genes, and a total of 12,722 regulating interactions were found.

**Fig. 3:**
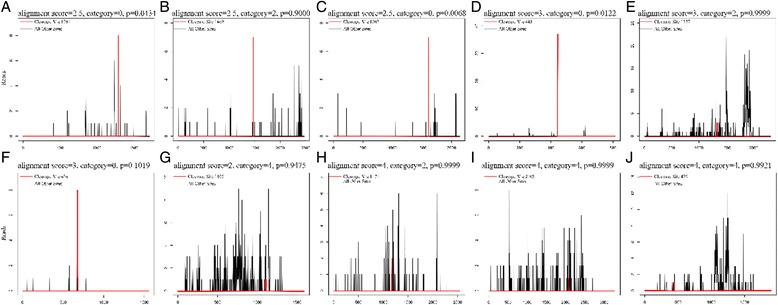
Target plot (t-plot) of representative validated miRNA targets in *P. nigra* × *P. deltoides* ‘Robusta.’ **a** miR172g-3p target to AP2/ERF superfamily transcription factor, cleavage site at 1291nt; **b** miR171i-p3 target to GRAS family transcription factor gene, cleavage site at 1469nt; **c** miR393_R + 2 target to F-box/RNI-like superfamily gene, cleavage site at 1607nt; **d** miR828a target to MYB transcription factor gene, cleavage site at 443nt; **e** miR172b-5p target to PtrWRKY70 gene, cleavage site at 1327nt; **f** miR482a target to TIR-NBS-LRR gene, cleavage site at 676nt; **g** miR5032-p3 target to GroES-like zinc-binding dehydrogenase family protein gene, cleavage site at 1105nt; **h** miR6485-p3 target to O-methyltransferases gene, cleavage site at 1171nt; **i** miR171e-p5 target to epsin N-terminal homology (ENTH) domain-containing protein gene, cleavage site at 2108nt; **j** miR6468 target to HXXXD-type acyl-transferase family gene, cleavage site at 439nt. The red line, which was enhanced with Photoshop CS6, indicates the cleavage site of each transcript

These targets included 31 plant defense genes that were regulated by 26 miRNA (Table 2), such as the disease resistance protein TIR-NBS-LRR, regulated by miR482a (Fig. 3f); the HXXXD-type acyl-transferase family protein gene, regulated by miR6468 (Fig. 3j); and the wound-responsive family protein, regulated by miR394-p3. Additionally, these targets included 27 transcription factors that were regulated by 27 miRNAs (Additional file 1: Table S3), such as the AP2/ERF superfamily transcription factor gene, regulated by miR172g-3p (Fig. 3a); the GRAS family transcription factor gene, regulated by miR171i-p3 (Fig. 3 b); the MYB transcription factor gene, regulated by miR828a (Fig. 3d); and the WRKY70 gene, regulated by miR172b-5p (Fig. 3e).

**Table 2:** Popular defense related genes responsive to rust infection and their regulating miRNA

Targets		miRNA		
Annomation	log_2_	miRNA	Group	log_2_
A20/AN1-like zinc finger family protein	1.16	miR855-p5	Gp3a	−0.4
Aldehyde dehydrogenase 3H1	−1.02	PC-5p-1903277_1	Gp4a	0.18
ARM repeat superfamily protein	2.55	miR172j-p3	Gp3b	10.06
Auxin response factor 16	1.06	miR160e-5p	Gp1a	0.25
BED zinc finger	0.55	miR858a-p3	Gp3b	10.06
Chaperone protein htpG family protein	−1.19	PC-3p-2241919_1	Gp4a	−10.88
Cysteine synthase D1	1.33	miR167a-p3	Gp3b	−9.88
Cytochrome P450, family 82, subfamily G, polypeptide 1	1.11	miR482b-p5	Gp3a	11.06
Disease resistance protein (TIR-NBS-LRR class)	−0.94	miR393b-p5	Gp3b	−9.88
Dormancy/auxin associated family protein	−1.29	miR396e-p3	Gp2b	10.06
Early nodulin-like protein 18	1.05	miR824-p3	Gp3a	2.18
ENTH domain-containing protein	0.54	miR171e-p5	Gp1a	1.77
F-box family protein	−0.65	miR394-p3	Gp3b	10.06
Glutaredoxin family protein	0.58	miR6300	Gp2b	−0.82
Glutaredoxin family protein	0.9	miR2606b-p3	Gp3a	0.18
Glutathione S-transferase 6	0.54	PC-3p-1909651_1	Gp4a	−9.88
Histone superfamily protein	1.37	miR400-p5	Gp3a	11.64
Homeodomain-like superfamily protein	−1.01	miR2655e-p3	Gp3b	−10.14
HXXXD-type acyl-transferase family protein	0.81	miR6468	Gp1a	−2.77
Jasmonate-zim-domain protein 1	−2.48	PC-3p-401772_2	Gp4a	−1.4
Late embryogenesis abundant domain-containing protein/LEA domain-containing protein	1.02	miR394-p3	Gp3b	10.06
myo-inositol-1-phosphate synthase 2	1.29	miR5072-p3	Gp3a	10.06
Pathogenesis-related 4	−1.02	miR5517-p5	Gp3a	1.18
P-loop containing nucleoside triphosphate hydrolases superfamily protein	1.04	miR394-p3	Gp3b	10.06
PR5-like receptor kinase	0.5	miR394-p3	Gp3b	10.06
Proton gradient regulation 5	−1.04	miR156g-p5	Gp3b	−9.88
RING/FYVE/PHD zinc finger superfamily protein	1.14	miR824-p3	Gp3a	2.18
TIR-NBS-LRR16	1	miR482a_2s	Gp2a	1.18
Trichome birefringence-like 33	1.1	miR5293-p5	Gp3a	−0.18
Wound-responsive family protein	−1.57	miR394-p3	Gp3b	10.06
WRKY DNA-binding protein 11	0.79	miR2097-p3	Gp3a	10.06

### Digital gene expression

To characterize the target gene expression pattern, two digital gene expression (DGE) libraries of -rust and + rust were constructed and sequenced. There were 16,409,618 and 18,186,321 reads sequenced from the -rust and + rust libraries, respectively. More than 99 % of the raw reads passed the filter cut-off, resulting in 18,117,197 and 16,338,785 clean reads. In total, 73,013 unique genes were obtained, and 65,822 of those unique genes were mappable. Of the unique reads, 1,331,698 (25.07 %) and 1,419,523 (24.85 %) sense reads were mapped to one gene for the -rust and + rust libraries, respectively. The remaining 73.38 % of the unique reads for the –rust library and the remaining 73.60 % for the + rust library matched to multiple genes. The remaining reads were unique antisense or both sense and antisense reads that were mappable to genes.

**Fig. 4:**
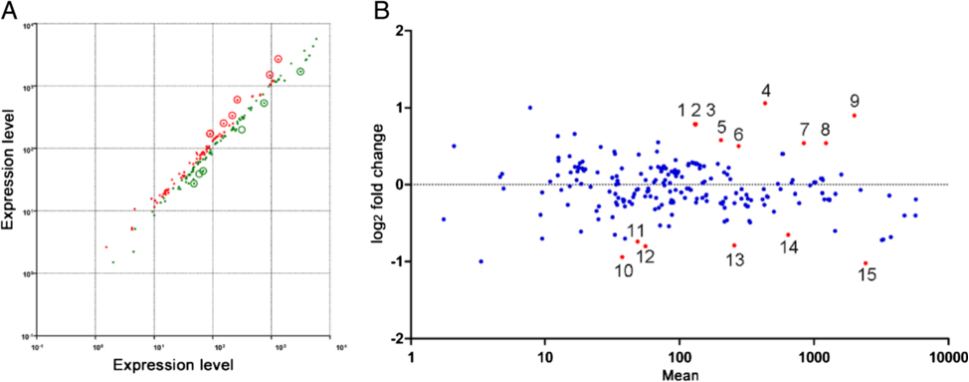
Scatter plot of rust infection-regulated gene expression. **a** Scatter plot of mRNA expression levels according the raw reads. Data points above (green) or below (red) the slope line represent down-regulated or up-regulated miRNAs. Differentially expressed genes are denoted with circles based on *x* ^2`^ (*P* ≤ 0.01). **b** Scatter plot of gene expression in + rust and –rust libraries according the log_2_ of normalized reads. Expression levels are normalized to TPM. Differentially expressed genes are labeled red based on *x* ^2^ (*P* ≤ 0.01) (details in Table 2). 1, 2 and 3: WRKY DNA-binding protein 11; 4: Auxin response factor 16; 5: Glutaredoxin family protein; 6: PR5-like receptor kinase; 7: epsin N-terminal homology (ENTH) domain-containing protein/clathrin assembly protein-related; 8: glutathione S-transferase 6; 9: Glutaredoxin family protein; 10: disease resistance protein (TIR-NBS-LRR class); 11: Histone H3 K4-specific methyltransferase SET7/9 family protein; 12: Disease resistance protein (TIR-NBS-LRR class); 13: HXXXD-type acyl-transferase family protein; 14: F-box family protein; 15: Pathogenesis-related 4

To compare the differential expression patterns between the -rust and + rust libraries, we normalized (by the means of TPM) the read distribution for the gene expression level in each library to construct an effective library size and determined the significance of differentially expressed transcripts with *x*^2^ (*P* ≤ 0.01). According to the annotation of these genes, the rust infection-regulated gene expression was analyzed (Fig. 4).

According to the results of the DGE analysis, the targets of the potential novel miRNAs with altered expression were analyzed (Tables 1 and Additional file 1: S4). Then, the log_2_-fold changes of plant defense-related genes and their regulating miRNAs were analyzed (Table 2).

On the basis of differential expression analysis [30] and control of the false discovery rate (FDR) [31], 282 differentially expressed target genes were identified. To compare these results with the real-time quantitative polymerase chain reaction (RT-qPCR) results, the log_2_- (+rust/-rust) fold changes >1 were designated as indicating ‘up-regulated genes’, and the changes < −1 were designated as indicating ‘down-regulated genes’.

Gene Ontology (GO) analysis was performed for all of the differentially expressed genes (Additional file 1: Table S5). Altogether, 282 differentially expressed target genes were linked to 56 GO terms. Once the GO terms of interest were identified for the unigenes, the differentially expressed genes and their associated miRNAs were analyzed (Additional file 1: Text S5). From the GO terms, we found that many processes, such as response to stress (GO:0006950), heat shock protein binding (GO:0031072), unfolded protein binding (GO:0051082), oxidation-reduction process(GO:0055114), were prominently down-regulated and that the processes of response to oxidative stress (GO:0006979), metabolic process (GO:0008152), biosynthetic process (GO:0009058), and cell wall macromolecule catabolic process (GO:0016998) were up-regulated.

### The impact of rust infection on post-transcriptional regulation in the susceptible poplar

**Fig. 5:**
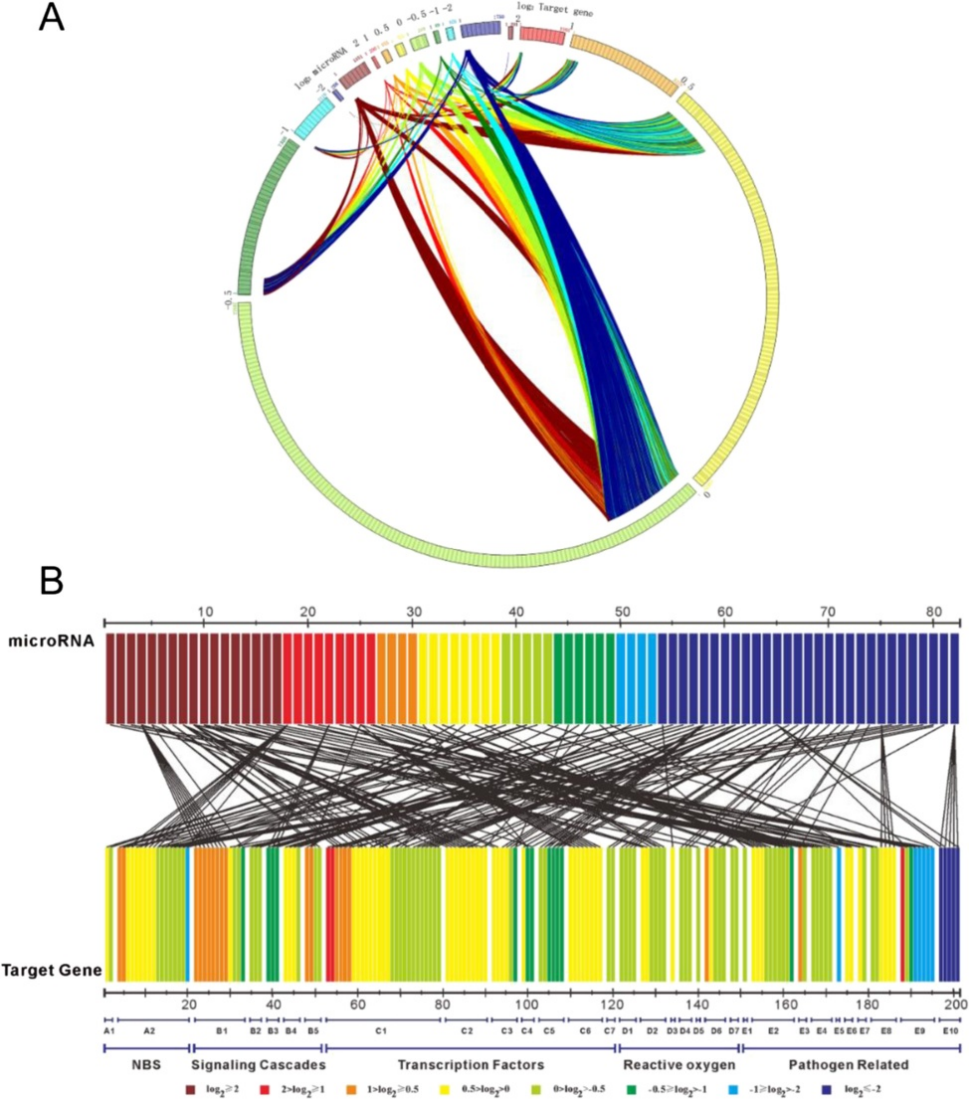
Post-transcriptional analyses of miRNAs and genes in response to infection by rust in ‘Robusta’. **a** Post-transcriptional analyses of miRNAs and genes in response to infection by rust in the susceptible poplar. **b** Post-transcriptional analyses of miRNAs and resistance genes in response to infection by rust in the susceptible poplar. A1: *CC-NBS-LRR*; A2: *TIR-NBS-LRR*; B1: *MAP kinase*; B2: *MAPK/ERK kinase*; B3: *Kunitz family trypsin and protease inhibitor protein*; B4: *2-cysteine peroxiredoxin*; B5: *Glutaredoxin family protein*; C1: *WRKY DNA-binding protein*; C2: *WRKY family transcription factor*; C3: *Basic-leucine zipper (bZIP) transcription factor family protein*; C4: *Ethylene responsive element binding factor*; C5: *MYB family transcription factor*; C6: *MYB-like HTH transcriptional regulator family protein*; C7: *Jasmonate-zim-domain protein*; D1: *Copper/Zinc superoxide dismutase*; D2: *Manganese superoxide dismutase*; D3: *Ascorbate peroxidase*; D4: *Monodehydroascorbate reductase*; D5: *Glutathione peroxidase*; D6: *Glutathione reductase*; D7: *Glutathione S-transferase*; D8: *Ferritin*; E1: *F-box and associated interaction domains-containing protein*; E2: *F-box family protein*; E3: *F-box/RNI-like superfamily protein*; E4: *Pathogenesis related homeodomain protein*; E5: *Pathogenesis-related protein*; E6: *Pathogenesis-related thaumatin superfamily protein*; E7: *Peptidoglycan-binding LysM domain-containing protein*; E8: *Phospholipase*; E9: *Wound-responsive family protein*; E10: *Wound-responsive protein-related*

**Fig. 6:**
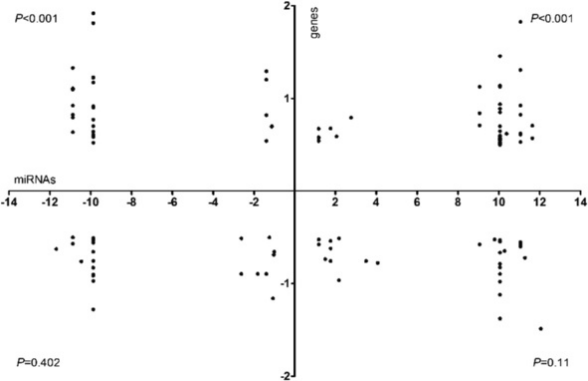
Targets of rust-responsive miRNAs among rust-regulated genes. The x-axis indicates the log_2_- (+rust/-rust) transformed fold change of rust-responsive miRNAs; log_2_- (+rust/-rust) fold changes >1 were designated as indicating ‘up-regulated miRNAs’, and changes < −1 were designated as indicating ‘down-regulatedmiRNAs’. The y-axis indicates the log_2_-transformed fold change of rust-regulated expressed genes; the log_2_- (+rust/-rust) fold changes >1 were designated as indicating ‘up-regulated target genes’, and changes < −1 were designated as ‘down-regulatedtarget genes’. The *P*-value was assessed by a 2-tailed *x* ^2^ test

From the analysis of the miRNA, degradome cDNA and DGE libraries, 12,722 regulatory interactions (947 miRNAs to 8,463 target genes) were identified (Fig. 5a). Among the 947 miRNAs involved, 250 down-regulated (log_2_-fold changes <1) and 301 up-regulated (log_2_-fold changes >1) rust-responsive miRNAs were associated with 4,099 and 4,781 regulatory interactions (Fig. 6), respectively. Among the 4,099 regulatory interactions targeted by 250 down-regulated miRNAs, 59 (1.439 %) target genes were up-regulated (log_2_-fold changes >1), 61 (1.488 %) target genes were down-regulated (log_2_-fold changes < −0.5), 3,967 (96.77 %) target genes were unchanged (log_2_-fold changes > −1 and <1), and the other target genes (12 regulating interactions) were undetected by DGE analysis. Among the 4,781 regulatory interactions targeted by 301 up-regulated miRNAs, 44 (0.92 %) target genes were down-regulated, 73 (1.53 %) target genes were up-regulated, 4,648 (97.22 %) target genes were unchanged, and the other target genes (16 regulating interactions) were undetected by DGE analysis. The resistance-related miRNAs and their target genes were found in poplar ‘Robusta’, but not all of those miRNAs and their target genes were in compliance with this negative regulation (Additional file 1: Figure S4, Additional file 1: Table S4, transcription model M2b).

Furthermore, we analyzed the post-transcriptional regulation of 82 miRNAs to 171 resistance genes, such as the genes encoding intracellular nucleotide-binding-site (NBS) -leucine-rich repeat (LRR) proteins (Fig. 5b, A1 and A2), signaling cascades (Fig. 5b, B1 to B5), transcription factors (Fig. 5b, C1 to C7), reactive oxygen (Fig. 5b, D1 to D7) and pathogenesis-related (Fig. 5b, E1 to E10) (Additional file 1: Table S4). Out of these 82 miRNAs, 28 (34 %) regulated only one target gene, whereas the other 54 (66 %) regulated more than one target gene. In contrast, 132 (77 %) resistance genes were regulated by one miRNA, whereas the remaining genes (23 %) were regulated by more than one miRNA.

Altogether, 226 resistance regulating interactions were identified, and these regulating interactions were further divided into five regulation modes. The first mode included 73 regulating interactions (32 %). The expression of target genes was unchanged (Additional file 1: Table S4, regulation mode M1b) or up-regulated (Additional file 1: Table S4, regulation mode M1a) in the first mode, although the accumulation of miRNA was increased. Only 9 regulating interactions (4 %) were identified in the second mode. In this mode, the accumulation of miRNA was unchanged, but the expression of target genes was up-regulated (Additional file 1: Table S4, regulation mode M2a) or strongly suppressed (Additional file 1: Table S4, regulation mode M2c). The maximum amount of regulating interactions, a total of 89 interactions (39 %), was identified in the third mode. In this mode, the accumulation of miRNA was decreased, but the expression of target genes was down-regulated (Additional file 1: Table S4, regulation mode M3c) or unchanged (Additional file 1: Table S4, regulation mode M3b). The fourth mode included 31 regulating interactions (14 %, Additional file 1: Table S4, regulation mode M2b), and both the miRNA and the target gene were unresponsive to the rust infection. The last mode, which is consistent with the classical post-transcriptional regulation theory, included 24 regulating interactions (11 %). In the last mode, the accumulation of miRNA and the expression of target genes were in compliance with negative correlation (Additional file 1: Table S4, regulation modes M1c and M3a).

### Validation and temporal pattern of miRNA expression and target genes

To avoid missing significant differential changes in the mixed samples sequencing, RT-qPCR was performed for 10 differentially expressed miRNAs (log_2_ > 1 or < −1) and target genes at 2 h post inoculation (hpi) of rust, 6 hpi, 12 hpi, 1 day post inoculation (dpi), 2 dpi, 4 dpi and 7 dpi. These miRNAs were selected based on principles Additional file 1: Text S5 and are listed in Additional file 1: Table S6. The expression patterns of the RT-qPCR results showed details that had not been found in the sRNA libraries and DGE sequencing.

**Fig. 7:**
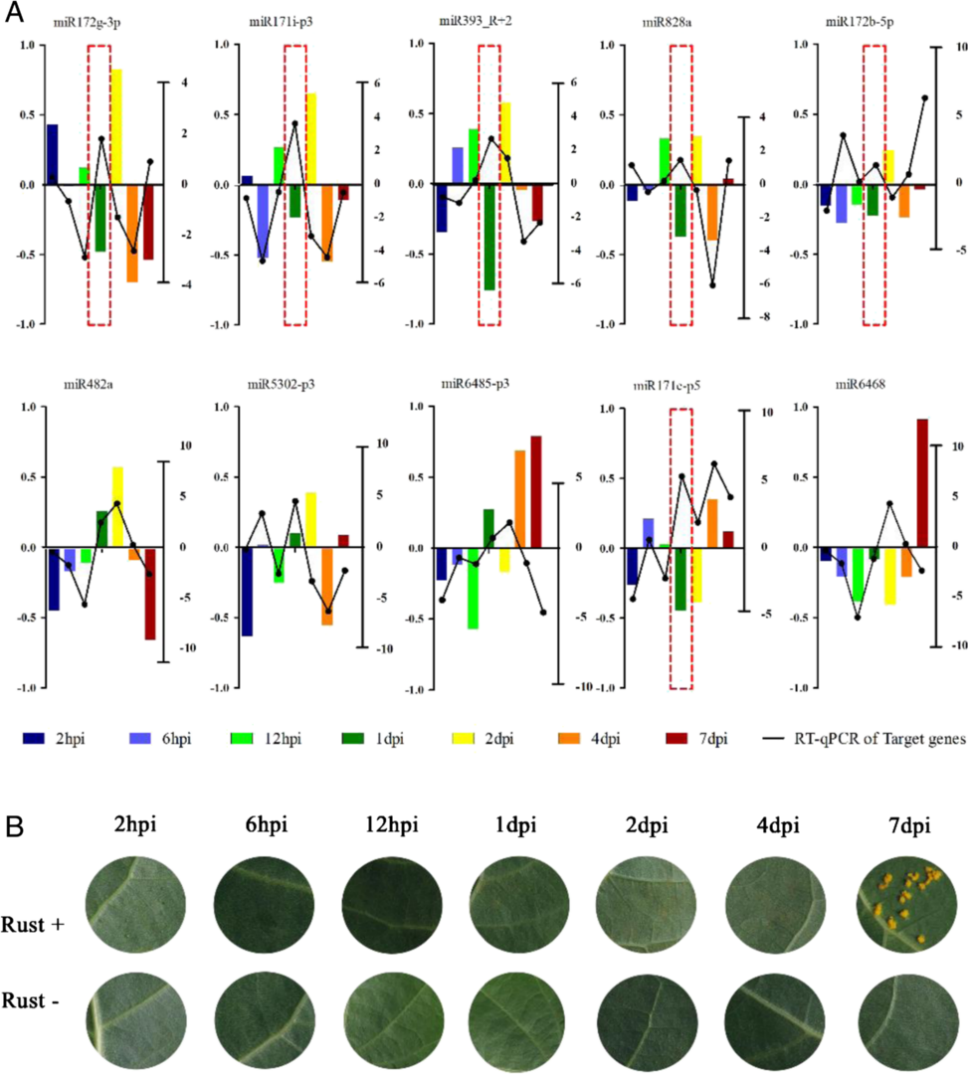
miRNA and target gene expression levels and symptoms at different time points post-inoculation. **a** Test of miRNA and target gene expression levels at different time points by RT-qPCR. **b** Symptoms at different time points post-inoculation. miR172g-3p: target to AP2/ERF superfamily transcription factor; miR171i-p3: target to GRAS family transcription factor gene; miR393_R + 2: target to F-box/RNI-like superfamily gene; miR828a: target to MYB transcription factor gene; miR172b-5p: target to PtrWRKY70 gene; miR482a: target to TIR-NBS-LRR gene; miR5032-p3: target to GroES-like zinc-binding dehydrogenase family protein gene; miR6485-p3: target to O-methyltransferases gene; miR171e-p5: target to epsin N-terminal homology (ENTH) domain-containing protein gene; miR6468: target to HXXXD-type acyl-transferase family gene. Error bars indicate SE (n = 3). Left-hand y-axes indicate the log_2_-fold change of miRNA; right-hand y-axes indicate the log_2_-fold change of the target gene

According to previous studies, before 1 dpi, infection hyphae extend into the mesophyll and differentiate into the first haustorial structures [32, 33]. Then, biotrophic growth continues, and the fungal biomass greatly increases between 2 and 4 dpi [34, 35]. During this time period, the expression of miRNA volatility continually changes over time, and we found 5 expression patterns related to the development of rust occurring on poplar leaves (Additional file 1: Figure S5; α, β, γ, δ and ε patterns, Additional file 1: Text S6). The expression of targets (Additional file 1: Figure S6) did not accompany changes in miRNA for all 7 time periods (Fig. 7), but several negative interactions were found for the α-pattern miRNA (miR172b-5p targeting the WRKY70 gene, miR171i-p3 targeting the GRAS family transcription factor gene, miR393_R + 2 targeting the F-box/RNI-like superfamily gene, miR828a targeting the MYB transcription factor gene and miR172b-5p targeting the WRKY70 gene) and the γ-pattern miRNA (miR171e-p5 targeting the epsin N-terminal homology domain-containing protein gene) at 1 dpi; in these cases, the targets were up-regulated, whereas the miRNAs were down-regulated (Fig. 7, boxed with red dashed lines).

### Framework of the susceptible poplar defense signal pathway

The miRNA and target global maps indicated that most of the related KEGG (Kyoto Encyclopedia of Genes and Genomes) pathways (Additional file 1: Figure S7) were down-regulated or up-regulated after rust infection, whereas few target genes were responsive to rust infection in the susceptible poplar. The other pathways, such as plant hormone signal transduction, cell cycle and plant-pathogen interaction, showed the same pattern; i.e., the miRNAs reacted strongly to the infection, but the target genes reacted mildly. The pathway of Plant-pathogen Interaction indicated that the miRNAs related to fungal pathogen-associated molecular patterns (PAMPs, including reactive oxygen species), Hypersensitive response (HR) and fungal effectors were responsive to rust infection. However, only 4 of 31 genes were up-regulated in the same pathway.

According to the regulatory interactions, an miRNA-mediated susceptible poplar defense signal pathway related to rust infection was revealed (Additional file 1: Table S7). The receptor-like kinase genes *RLK 25*, *RLK 26*, *RLK 29*, *RLK 34* and *RLK 42* were up-regulated, whereas the regulating miRNAs were down-regulated by rust infection. *RLK 14* was up-regulated, but the regulating miRNAs remained unchanged. *RLK 3* was up-regulated, as were the regulating miRNAs up-regulated.

The NBS-LRR family of resistance (R) proteins induced effector-triggered susceptibility (ETS). The results of degradome cDNA library sequencing indicated that 25 TIR-NBS-LRR family genes were regulated by 9 miRNAs. Although 7 miRNAs were down-regulated significantly, none of the corresponding 18 target genes were up-regulated. Two miRNAs were up-regulated, whereas the target TIR-NBS-LRR family genes were unchanged. Only 1 TIR-NBS-LRR gene family was up-regulated after the infection of rust fungi, whereas the regulating miRNA was unchanged. Additionally, 4 unchanging CC-NBS-LRR family genes were regulated by 2 down-regulated miRNAs. The suppression of the TIR-NBS-LRR-regulating miRNA indicated that recognition occurred between the rust effectors and the poplar R proteins but that the signal promoting the expression of R proteins was blocked. This process explains why ‘Robusta’ cannot prevent rust infection at the ETS stage.

Many downstream defense signal pathways were also found. First, pathogenesis-related (PR) genes, which were triggered by effector signals and transcription factors, were investigated (Additional file 1: Table S7). MiR5517-p5 was up-regulated, and its target PR gene was suppressed. Two pairs of PR genes and their regulating miRNA were unchanged. MiR3447-p3 was strongly up-regulated, yet its target PR gene was unchanged.

Second, the pathway related to salicylic acid (SA), which is the main signal molecule for systemic acquired resistance (SAR) and HR, did not respond to rust infection, although many of the regulating miRNAs were found to be down-regulated or up-regulated after infection. The *enhanced disease susceptibility 1* gene (*EDS1*, regulated by the promotion of miR3434-p3) and the *phytoalexin-deficient 4* gene (*PAD4*, regulated by the suppression of miR4248b-p3), which act to induce genes upstream of SA accumulation, did not respond to rust infection. Ankyrin-repeat protein NPR1, which acts downstream of SA and promotes the expression of the SAR-associated genes *PR-1*, *BGL2*, and *PR-5* in *Arabidopsis* [36], also did not respond to rust infection. The expression of the ‘Robusta’ *enhanced disease susceptibility 5* (*EDS5*) gene was up-regulated slightly (it is also regulated by the suppression of miR4248b-p3), whereas *SA induction–deficient 2* (*SID2*) was unchanged (regulated by the up-regulated PC-5p-1150271_1) after rust infection, although *EDS5* and *SID2* function upstream of SA accumulation in *Arabidopsis* [37]. The programmed cell death-related gene also did not respond to rust infection. These results suggest that the signal transduction pathways are blocked, which typically leads to an HR-related accumulation of SA and programmed cell death to halt pathogen invasion. Third, with the exception of the suppression of *mitogen-activated protein kinase 4* (*MPK4*, regulated by the down-re down-regulated PC-3p-272434_3) down-regulated and the promotion of *12-oxpophytodienoic acid reductase 3* (*OPR3*, regulated by the down-regulated PC-5p-1312398_1) up-regulated, no genes related to jasmonic acid (JA) or ethylene (ET) responded to rust infection, including *coronatine-insensitive protein 1* (*COI1*, undetected), *jasmonic acid-amido synthetase 1* (*JAR1*, regulated by the down-regulated miR393b-p5_1) and *Ethylene-insensitive protein 2* (*EIN2*, undetected). Normally, JA- and ET-dependent resistance is up-regulated by lesion formation in *A. thaliana*. MPK4, COI1 and JAR1 are required to transduce the JA signal, whereas EIN2 is required to transduce the ET signal [36].

## Discussion

The miRNA-mediated post-transcriptional regulation of plant resistance to biotic stress has been described in several plant pathosystems [22, 36–41]. In contrast, the molecular mechanisms of plant susceptibility to biotic stress and the post-transcriptional regulation of such susceptibility are poorly studied. Identifying the genetic basis of host susceptibility/resistance is a prerequisite for understanding the interaction between pathogen and plant host. Based on miRNA, degradome cDNA and DGE library analyses, we investigated the regulatory functions of miRNA in a susceptible poplar under rust attack. We found that the genes involved in the miRNA-mediated post-transcriptional regulation of the defense signal pathway were inactivated after infection by E4 in ‘Robusta’, whereas this regulation was functional during E1 infection. This inactivation was the major characteristic of ‘Robusta’ susceptibility.

The small RNA sequencing revealed a significant peak in abundance of 15-nt reads, with 835 unique 15-nt miRNAs were found in the + rust sRNA library. Moreover, 120 miRNAs targeted 113 unigenes, which included many disease-related genes, such as the AP2/B3 transcription factor protein family, the disease resistance CC-NBS-LRR class protein family, the disease resistance TIR-NBS-LRR class protein family, cellulose synthase 6 genes, LRR and NB-ARC domain-containing disease-resistance protein genes and stress-inducible protein genes. However, unexpectedly, none of these disease-related target genes responded to rust infection. These results indicate that the 15-nt miRNAs responded to rust infection and showed a significant increase but that they failed to regulate their target genes in the susceptible poplar.

Basal defense and *R* gene-mediated resistance are the two branches of the defense response carried out by plants under pathogen stress [42]. Basal defense marks the first line of defense and consists of a set of defined receptors referred to as pattern recognition receptors (PRRs), which recognize conserved, slowly evolving MAMPs or PAMPs [44]. The receptor-like kinases, which are part of the initial stages of PAMPs and PTI, were up-regulated in this compatible (susceptible poplar *vs.* virulent rust) pathosystem, and the miRNAs related to these stages were responsive to rust infection. These results indicate that the poplar defense commences with pathogen-associated molecular patterns (PAMPs) and PAMP-triggered immunity (PTI) and that the miRNAs are still functional at this stage.

The second branch of the defense response acts largely inside the cell and uses the polymorphic NB-LRR protein products encoded mostly by *R* genes [44]. Although the basal defense of the poplar was almost completely destroyed in this compatible pathosystem, *R* gene-mediated resistance was not fully functional. Moreover, the post-transcriptional regulation of the *R* gene did not occur. Only 1 TIR-NBS-LRR gene family, regulated by miR472b, was up-regulated after rust fungi infection, although poplar genomes contain 64 TIR-NBS-LRR and 119 CC-NBS-LRR immune receptors encoded by *R* genes that recognize the pathogen infection and trigger resistance responses [45].

Twelve miRNAs that guide the cleavage of 4 *CC-NBS-LRR* and 25 *TIR-NBS-LRR* transcripts were identified (Additional file 1: Table S7). Two *TIR-NBS-LRR* and 2 *CC-NBS-LRR* genes were regulated by miRNAs that belong to the mir482 superfamily; this result is consistent with those from previous studies [46, 47]. Both miR482b and its target *CC-NBS-LRR* were unresponsive to rust infection. In contrast, although miR482 was up-regulated (log_2_-fold change =1.77), 6 *TIR-NBS-LRR* target genes were unchanged (log_2_-fold change values ranging from 0.04 to 0.49). As one of the most important plant immune system branches [4], NBS-LRR-mediated disease resistance is effective against obligate biotrophic pathogens and hemi-biotrophic pathogens [48]. Our results showed that the susceptible poplar NBS-LRR-related genes were not up-regulated by the rust biotrophic pathogen. Interestingly, another study showed that NBS-LRRs are not substantially induced by an incompatible rust strain in *P. trichocarpa* × *P. deltoides* [45]. These findings suggest that the lack of substantial induction of NBS-LRRs by infection is not a characteristic specific to susceptible poplar but is common to both susceptible and resistant poplars.

All of the signaling component genes, including the genes related to *ET*, *SA, JA* and *HR,* were unresponsive to rust infection, which suggests that the regulation of miRNA-based HR signaling was blocked. Normally, plants produce phytohormones to cope with infection by diverse pathogens [49]. SA is involved in resistance to biotrophic pathogens, whereas JA and ET mediate resistance primarily to necrotrophic pathogens [50]. These results are consistent with our expectations and the gene-for-gene hypothesis, because the heterozygous resistant (*Rr*) poplar and the homozygous virulent (*avr*) rust were used in this study. Most of the studied plant pathosystems are related to the *R-Avr* gene-mediated disease response during pathogen invasion [42]. However, in our study, the plant pathosystem of susceptible poplar vs. virulent rust (an *R-avr* gene-mediated disease response) provides a new perspective for understanding plant resistance/susceptibility. However, our findings provide only initial insight into this topic, and a great effort will be required for a full understanding of the post-transcriptional regulation related to plant resistance/susceptibility.

## Conclusions

The results of the sRNA, degradome cDNA and DGE library analyses indicated that the defense-related post-transcriptional regulation of the susceptible poplar ‘Robusta’ only function normally during the initial stages of PAMPs and PTI. More importantly, the miRNA-mediated post-transcriptional regulation of defense signal pathway genes were inactivated after infection by virulent rust at the stage of effector-triggered susceptibility and at the subsequent stages of salicylic acid and HR response. This inactivation is the major characteristic of ‘Robusta’ susceptibility.

## Methods

### Rust isolates

The filial generation of virulent E4 and avirulent E1 was used in this study. Rust-infected poplar leaves were collected from *P. trichocarpa* cv. *trichobel*at Markington (northern England) and Alice Holt, Surrey (southern England). Rust isolates were derived from single uredinial pustules according previously reported methods [4]. The rust spores were stored at −20 °C.

### Plant materials and inoculation procedure

The 1-year-old dormant cuttings of the hybrid poplar (*P. nigra* × *P. deltoides*) ‘Robusta’ were used as a source of plant tissue. ‘Robusta’ was chosen because a previous study has found that this cultivar showed non-race-specific resistance to *M. larici-populina* isolates collected from England [4].

‘Robusta’ plants were grown in pots containing a sand-peat (50:50, v/v) mixture and were watered daily with deionized water under 16-h/8-h photoperiod in greenhouse conditions and 60-70 % relative humidity for 10 weeks. At the time of inoculation, young trees were 70–100 cm tall and bore 10 to 14 fully expanded leaves.

The inoculation procedure was performed as described by Pei *et al*. [4] with some modifications. In brief, fully expanded leaves from leaf plastochrony index (LPI) 5 to 9 were detached from ‘Robusta’ plants and spray-inoculated on their abaxial surface with a rust spore suspension in deionized water containing 0.004 % Tween 20 (1 drop in 100 ml) adjusted to 100,000 spores ml^−1^, or with deionized water containing 0.004 % Tween 20 as a control. After inoculation, the inoculated leaves were incubated in a growth chamber at 16 °C with 16 h day^−1^ illumination (80 uE m^−2^ s^−1^) for various durations. Each treatment was replicated three times. The samples harvested at different time points (2 hpi, 6 hpi, 12 hpi, 1 dpi, 2 dpi, 4 dpi, and 7 dpi) in the different treatments were then immediately snap-frozen in liquid nitrogen and maintained at −80 °C for further nucleic acid isolation.

### Total RNA preparation

Total RNA was extracted from frozen leaves of ‘Robusta’ with the CTAB method. Two sets of total RNA were prepared, with one derived from the original RNA pool prepared from E4 rust-inoculated leaves (+rust) at the 2 hpi, 6 hpi, 12 hpi, 1 dpi, 2 dpi, 4 dpi, and 7 dpi time points and the other from the RNA pool derived from rust-free leaves (−rust, the control) at the same time points.

### sRNA, degradome cDNA and DGE library preparation and sequencing

Two sRNA libraries were generated from the -rust and + rust RNA pools using the Illumina TruSeq Small RNA Preparation Kit according to Illumina’s TruSeq Small RNA Sample Preparation Guide 1.

The degradome cDNA library of + rust was prepared following procedures previously described (Additional file 1: Text S7) [48,51-53]. Two DGE libraries representing -rust and + rust were prepared according to the Illumina/Solexa standard protocol (directional mRNASeq sample preparation part #15018460 Rev. A, October 2010. Illumina, San Diego, CA, USA) (Additional file 1: Text S8).

The purified sRNA, degradome cDNA and DGE libraries were used for cluster generation on Illumina’s Cluster Station and then sequenced on the Illumina GAIIx platform by LC Sciences (Houston, TX, USA). To analyze the differential expression, three biological replicates were performed with the sRNA, degradome cDNA and DGE libraries. Each sample of degradome cDNA was sequenced for 80 cycles on one lane of the Illumina GAIIx platform (Illumina, San Diego, CA, USA) with the 2 × 100 bp module. DGE libraries were sequenced using the 1 × 36 bp module.

Raw sRNA library sequencing reads were obtained using Illumina’s Sequencing Control Studio software version 2.8 (SCS v2.8) following real-time sequencing image analysis and base-calling by Illumina’s Real-Time Analysis version 1.8.70 (RTA v1.8.70).

Raw sequencing reads of the degradome cDNA library were obtained using Illumina’s Pipeline version 1.5 software following sequencing image analysis by the Pipeline Firecrest Module and base-calling by the Pipeline Bustard Module.

### Bioinformatics analysis

A proprietary pipeline script, ACGT101-miR version 4.2 (LC Sciences, Houston, TX, USA), was used for the sRNA library sequencing data analysis to obtain mappable sequences from raw sequencing data and to map the miRNA-mappable unique sequences to pre-miRNA, mature miRNA or genome.

A global normalization was used to correct sRNA copy numbers among the -rust and + rust libraries (Additional file 1: Text S10). The differential gene expression of the DGE libraries was analyzed according to the method of Simon Anders, [30].

### RT-qPCR of mature miRNAs

RT-qPCR was performed with the One Step Prime-Script miRNA cDNA Synthesis Kit and the SYBR Premix ExTag II reagent kit (TaKaRa, Dalian, China) using the Stratagene Mx3000P qPCR system (Agilent, USA) to validate the expression levels of miRNAs after the rust inoculation at 2 hpi, 6 hpi, 12 hpi, 1 dpi, 2 dpi, 4 dpi and 7 dpi. All of the primers used in this study are listed in Additional file 1: Table S8. The 5.8S ribosomal RNA was used as the internal control [54]. This experiment was performed on three biological replicates. Quantification of the RT-qPCR results was conducted as previously described [55]. To compare the RT-qPCR results with the results of the high-throughput sequencing, the RT-qPCR results were normalized as log_2_- (+rust miRNA copy/-rust miRNA copy) fold changes.

## Availability of supporting data

All the supporting data are included as additional files as “Additional file 1”.

## Additional file

Additional file 1: Text S1. Unique miRNA classification. **Text S2**. Eleven features of miRNAs hairpin. **Text S3**. Five categories of the sliced-target transcripts. **Text S4**. Gene Ontology (GO) analysis. **Text S5**. Principles of the miRNA selected for RT-qPCR. **Text S6**. The 5 expression patterns of miRNA volatility change related to the development of rust. **Text S7**. Methods of + rust degradome cDNA libraries construction. **Text S8**. Detailed methods of RNAseq libraries construction. **Text S9**. The functions of CleaveLand 3.0 used in this study. **Text S10**. Procedure of a modified global normalization. **Figure S1**. Pie plots of data filtering and database mapping of the sRNA libraries from *P. nigra* × *deltoids* infected (+rust) and uninfected (−rust) with rust fungi *M. larici-populina.* **Figure S2**. Length distribution of mappable reads in sRNA libraries from *P. nigra* × *deltoids* infected (+rust) and uninfected (−rust) with rust fungi *M. larici-populina.* **Figure S3**. Predicted secondary structures of potential novel miRNAs from *P. nigra* × *deltoids*. Sequences indicated in red correspond to predicted miRNA. **Figure S4**. Targets of rust-responsive miRNAs among rust-regulated expressed genes. **Figure S5**. miRNA expression level validated by RT-qPCR at different time points post inoculation. **Figure S6**. Target gene expression level validated by RT-qPCR at different time points post inoculation. **Figure S7**. KEGG pathway and atlas. **Table S1**. A summary of standard data analysis results of the sRNA libraries from *P. nigra* × *deltoids* ‘Robusta’ infected (+rust) and uninfected (−rust) with rust fungi *M. larici-populina.* **Table S2**. Data summary of degradome library. **Table S3**. Transcription factors and their regulating miRNA. **Table S4**. post-transcriptional analyses of miRNA and resistance genes response to the infection of rust in ‘Robusta’. **Table S5**. The Gene Ontology of the rust-responsive gene and their regulating miRNA. **Table S6**. miRNA and target genes expression level and symptoms at different time points post inoculation. **Table S7**. Poplar defense pathway miRNA and target genes. **Table S8**. Primers used in the RT-qPCR. (DOC 3467 kb)

## Abbreviations

avr: Homozygous virulent
COI1: Coronatine-insensitive protein 1
DGE: Digital gene expression
dpi: Days post inoculation
EDS1: Enhanced disease susceptibility 1
EDS5: Enhanced disease susceptibility 5
EIN2: Ethylene-insensitive protein 2
ET: Ethylene
ETS: Effector-triggered susceptibility
GO: Gene Ontology
hpi: Hours post inoculation
HR: Hypersensitive response
JA: Jasmonic acid
JAR1: Jasmonic acid-amido synthetase 1
KEGG: Kyoto Encyclopedia of Genes and Genomes
LPI: Leaf plastochrony index
LRR: Leucine-richrepeat
miRNAs: MicroRNAs
NBS: Nucleotide-binding-site
PAD4: Phytoalexin-deficient 4
PAMPs: Pathogen-associated molecular patterns
PR: Pathogenesis-related
PRRs: Pattern recognition receptors
PTI: The PAMP-triggered immunity
R: Resistance proteins
Rr: Heterozygous resistant
RT-qPCR: Quantitative real time polymerase chain reaction
SA: Salicylic acid
SAR: Systemic acquired resistance
sRNA: small RNA
