# Supplementary material for: MicroRNA-mediated susceptible poplar gene expression regulation associated with the infection of virulent Melampsora larici-populina
Source: BMC Genomics. 2016 Jan 15;17:59. doi: 10.1186/s12864-015-2286-6 (PMC4714501; doi:10.1186/s12864-015-2286-6)
Supplement: Additional file 1: Text S1. — Unique miRNA classification. Text S2. Eleven features of miRNAs hairpin. Text S3. Five categories of the sliced-target transcripts. Text S4. Gene Ontology (GO) analysis. Text S5. Principles of the miRNA selected for RT-qPCR. Text S6. The 5 expression patterns of miRNA volatility change related to the development of rust. Text S7. Methods of + rust degradome cDNA libraries construction. Text S8. Detailed methods of RNAseq libraries construction. Text S9. The functions of CleaveLand 3.0 used in this study. Text S10. Procedure of a modified global normalization. Figure S1. Pie plots of data filtering and database mapping of the sRNA libraries from P. nigra × deltoids infected (+rust) and uninfected (−rust) with rust fungi M. larici-populina. Figure S2. Length distribution of mappable reads in sRNA libraries from P. nigra × deltoids infected (+rust) and uninfected (−rust) with rust fungi M. larici-populina. Figure S3. Predicted secondary structures of potential novel miRNAs from P. nigra × deltoids. Sequences indicated in red correspond to predicted miRNA. Figure S4. Targets of rust-responsive miRNAs among rust-regulated expressed genes. Figure S5. miRNA expression level validated by RT-qPCR at different time points post inoculation. Figure S6. Target gene expression level validated by RT-qPCR at different time points post inoculation. Figure S7. KEGG pathway and atlas. Table S1. A summary of standard data analysis results of the sRNA libraries from P. nigra × deltoids ‘Robusta’ infected (+rust) and uninfected (−rust) with rust fungi M. larici-populina. Table S2. Data summary of degradome library. Table S3. Transcription factors and their regulating miRNA. Table S4. post-transcriptional analyses of miRNA and resistance genes response to the infection of rust in ‘Robusta’. Table S5. The Gene Ontology of the rust-responsive gene and their regulating miRNA. Table S6. miRNA and target genes expression level and symptoms at different time points post inoculation. Table S7. P [file 12864_2015_2286_MOESM1_ESM.doc]

**MicroRNA-mediated susceptible poplar gene expression regulation associated with the infection of virulent *Melampsora larici-populina***

Danlei Li^§^, Feng Wang^§*^, Chao Wang, Li Zou, Zhiying Wang^*^, Qiaoli Chen, Chunyang Niu, Ruizhi Zhang, Yaming Ling, Bowen Wang

College of Forestry, Northeast Forestry University, Harbin, China

**^§^**These authors contributed equally to this work

^*^Corresponding authors: Zhiying Wang, College of Forestry, Northeast Forestry University, Harbin 150040, China; Tel: +86-451-82190384, E-mail: [nematodefungi@126.com](mailto:nematodefungi@126.com); Feng Wang, College of Forestry, Northeast Forestry University, Harbin 150040, China, Tel: +86-451-82190384, E-mail: [fengwang@](mailto:fengwang@)nefu.edu.cn

**Supplemental data files**

**Text S1 Unique miRNA classification**

Those unique miRNA were classified into two groups: known miRNAs and predicted miRNAs (Figure S1 C and D, Table S1). Known miRNAs were further divided into two subgroups: Gp1a (known mature miRNAs of *P.* *nigra* or *P. deltoids*) and Gp1b (known mature miRNAs of plant, but novel to *P. nigra* and *P. deltoids*). Predicted miRNAs were further divided into six subgroups: Gp2a (mapped to known Pre-miRNA and genome of other plants, within hairpins), Gp2b (mapped to known Pre-miRNA and genome of other plants, no hairpins), Gp3a (mapped to known Pre-miRNA and mature miRNAs of other plants, but unmapped to poplar genome), Gp3b (mapped to known Pre-miRNA of other plants, but unmapped to poplar genome), Gp4a (unmapped to known mature miRNAs, but mapped to genome and within hairpins) and Gp4b (unmapped to known mature miRNAs, no hairpins). By removing the no hairpin subgroups (Gp2b, Gp3b and Gp4b) and the subgroups unmapped to poplar genome (Gp3a and Gp3b), Gp1a, Gp1b, Gp2a and Gp4a were named as candidate miRNA subgroups. In terms of the candidate miRNA subgroups, 1,474 (Gp1a: 442; Gp1b: 21; Gp2a: 91; Gp4a: 920) and 1,475 (Gp1a: 438; Gp1b: 21; Gp2a: 76; Gp4a: 940) miRNA were obtained from -rust and +rust sRNA libraries respectively.

**Text S2 Eleven features of miRNAs hairpin ( LC Sciences)**

1. number of allowed errors in one bulge in stem: <= 12

2. number of basepair (bp) in stem region: >= 16 bp (in stem)

3. free energy (dG in kCal/mol): <=-15

4. length of hairpin (up and down stem + terminal loop): >= 50

5. length of terminal loop: <= 20

6. number of allowed errors in one bulge in mature region: <= 8

7. number of allowed biased errors in one bulge in mature region: <= 4

8. number of allowed biased bulges in mature region: <= 2

9. number of basepair (bp) in mature or mature* region: >= 12

10. percentage of small RNA in stem region (pm): >= 80%

11. number of allowed errors in mature region: <=7

12. MFEI>= 0.7, Definition of MFEI: MFEI = -dG*100/mirLen/CG%.

**Text S3 Five categories of the sliced-target transcripts**

Category ‘0’ is defined as > 1 raw read at the position, with abundance at a position equal to the maximum on the transcript, and with only one maximum on the transcript. Category ‘1’ is described as > 1 raw read at the position, with abundance at the position equal to the maximum on the transcript, and more than one maximum position on the transcript. Category ‘2’ includes > 1 raw read at the position, and abundance at the position less than the maximum but higher than the median for the transcript. Category ‘3’ comprised the transcripts with > 1 raw read at the position, and abundance at the position equal to or less than the median for the transcript; and category ‘4’ showed only one raw read at the position.

**Text S4** **Gene Ontology (GO) analysis**

Differentially expressed genes were classified into biological processes (34.2%), cellular component (11.7%) and molecular function (54.8%) (Figure S5). Briefly, most of the differentially expressed genes were associated with binding and catalytic activity processes. ATP binding, protein binding, zinc ion binding, heat shock protein binding, unfolded protein binding and sequence-specific DNA binding were the most expressed ontologies in the binding process. Protein kinase activity, oxidoreductase activity, catalytic activity, inositol-3-phosphate synthase activity and ubiquitin-protein ligase activity were the most expressed ontologies in the catalytic activity process.

**Text S5 Principles of the miRNA selected for RT-qPCR**

RT-qPCR of 10 differential expression miRNAs were performed. Those 10 miRNAs were selected based on principles:

1. Only the miRNAs of candidate subgroups (Gp1a, Gp1b, and Gp2a) were selected;
2. Only miRNAs with more than 5 raw reads in both of the two sRNA libraries were selected;
3. Log_2_(+rust/-rust) fold changes of the miRNA copy numbers were >1 or < −1 in the sRNA libraries sequencing;
4. The targets of the miRNA could be found, and the target gene expression pattern could be found in the DGE libraries;
5. The targets of the miRNA could be functional annotated;
6. Only miRNAs with one target gene were selected.

**Text S6 The 5 expression patterns of miRNA volatility change related to the development of rust**

In α patterns, suppression of miRNAs indicated that their target gene should be promoted at the time of haustorial formation (1dpi). Then, a sharp reversal promoted miRNA expression was found at the time of biotrophic growth beginning (2dpi). This result indicated that the susceptible poplar was conquered by the rust in the compatible interaction. Soon, a sharp reversal suppression was found at 4dpi. Although a sharp reversal suppression was found at 4dpi, the fight back of the poplar would not affect the fact that the long-term biotrophic feeding relationship was established. Thereafter, seesaw battles between poplar and rust would continue, as shown by different miRNA expression patterns. Similar to α expression pattern, seesaw battles were found in the β expression. The characteristics of the β expression pattern were that the regular suppression of miRNAs begin at 12hpi, and had an extended fight back time (from 2dpi to 4dpi). The γ and δ expression patterns were diametrically opposite to α and β expression patterns, respectively. In the ε expression pattern, a sharp reversal promoted miRNA expression was found at the time of 7dpi.

**Text S7 Methods of degradome cDNA libraries construction**

Construction of degradome libraries differed considerably from past efforts with some modification.

1. Approximately 150 ng of poly(A)+ RNA was used as input RNA and annealing with Biotinylated Random Primers.
2. Strapavidin capture of RNA fragments through Biotinylated Random Primers.
3. 5’ adaptor ligation to to only those RNAs containing 5’-monophosphates.
4. Reverse transcription and PCR
5. Libraries were sequenced using the 5’ adapter only, resulting in the sequencing of the first 36 nucleotides of the inserts that represented the 5’ ends of the original RNAs.

**Text S8 Detailed methods of RNAseq libraries construction**

Polyadenylated RNA was enriched by two rounds of poly-A selection with poly-T oligo-attached magnetic beads. The RNA was then chemically fragmented, treated with Antarctic phosphatase (NEB, Herts, UK) and subsequently with polynucleotide kinase (NEB, Herts, UK). V1.5 sRNA 3`Adapter was ligated to the RNA with T4 RNA ligase 2 truncated (NEB, Herts, UK), and SRA 5` Adapter was ligated to the RNA with T4 RNA Ligase. After ligation of the SRA RT primer, the RNA was reverse transcribed with SuperScript II Reverse Transcriptase (Invitrogen, Carlsbad, CA, USA). Double stranded sequencing library DNA was then produced by 15 cycles PCR with primers GX1 and GX2. The PCR products from 200bp to 300bp were recycled by 6% polyacrylamide Tris-borate-EDTA gel.

**Text S9 The functions of CleaveLand 3.0 used in this study**

1. Get degradome data, free from adapter sequences, etc.
2. Map the degradome reads to the appropriate transcriptome using appropriate thresholds.
3. Filter the mapped data, if required (see notes below).
4. Summarize the mapped degradome data into a "degradome density file" using script 'CleaveLand3_map2dd.pl'.
5. Generate small RNA / mRNA alignments using 'targetfinder.pl'.
6. Compare the degradome density file to the target predictions, and output significant hits using script 'CleaveLand3_analysis.pl'.
7. Generate "t-plots" of the targets using script 'CleaveLand3_t-- plotter.pl'.

**Text S10 Procedure of a modified global normalization**

1. Find a common set of sequences among all samples
2. Construct a reference data set. Each data in the reference set is the copy number median value of a corresponding common sequence of all samples.
3. Perform 2-based logarithm transformation on copy numbers log_2_(copy)of all samples and reference data set. Δlog_2_(copy)
4. Calculate the log_2_(copy) difference Δlog_2_(copy) between individual sample and the reference data set.
5. Form a subset of sequences by selecting|Δlog_2_(copy)|<2, which means less than (22=) 4 fold change from the reference set.
6. Perform linear regressions between individual samples and the reference set on the subset sequences to derive linear equations iy=a_i_x+b_i_， where ia and ib are the slop and interception, respectively, of the derived line, log_2_(copy)copy of the reference set, and y is the expected l log_2_(copy)copy of sample i on a corresponding sequence.
7. Calculate the mid value x_mid_=(max(x)-min(x))/2 of the reference set. Calculate the corresponding expected log_2_(copy)copy of sample i, yi, mid= aixmid+bi. Let y_r,mid_=x_mid_, let Δy_i_, = y_r,mid_-y_i,mid_, which is the logarithmic correction factor of sample i. We then derive the arithmetic correction factor fi = 2^Δyi^ sample i.
8. Correct copy numbers of individual samples by multiplying corresponding arithmetic correction factor *fi* to original copy numbers.


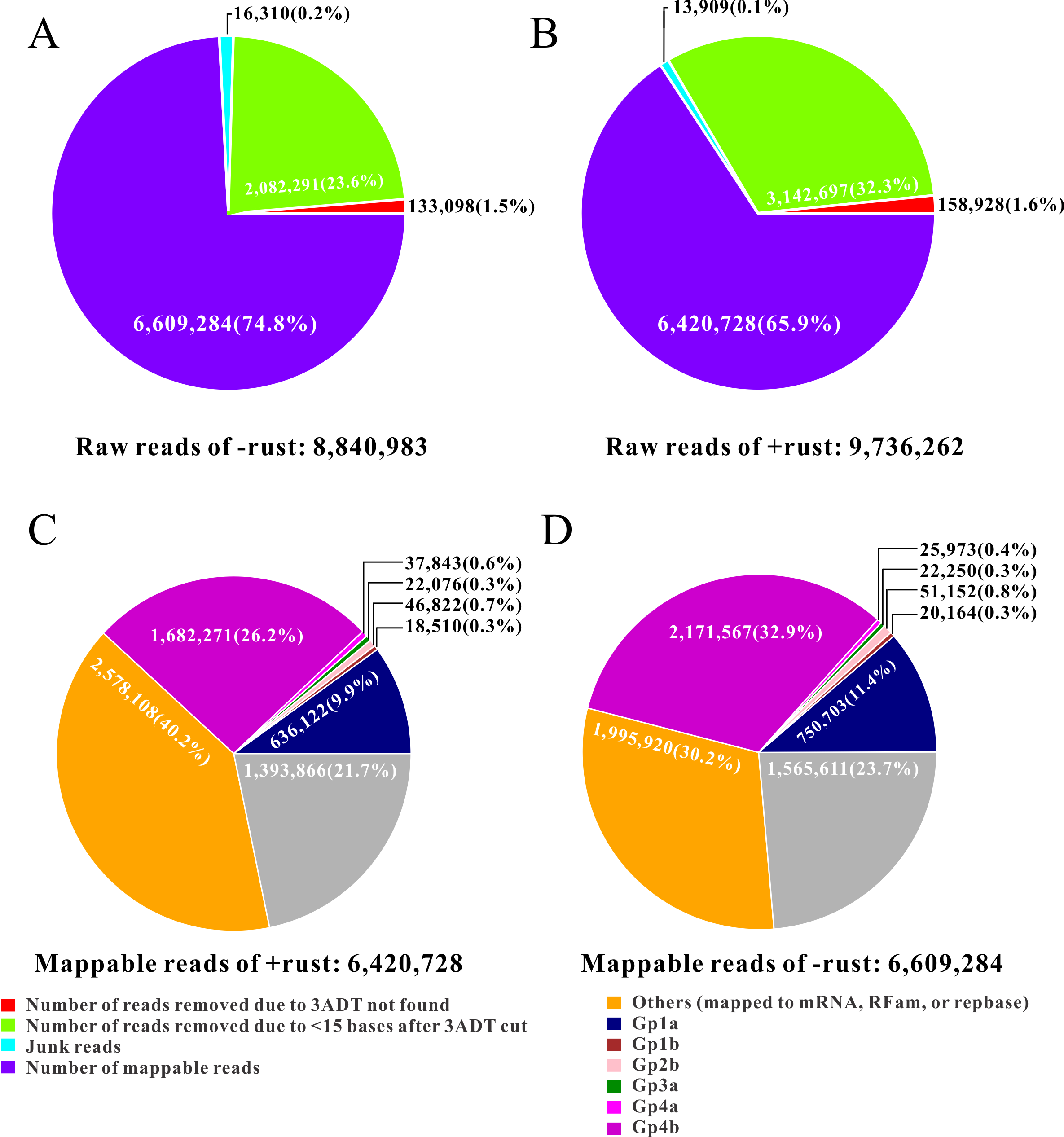


Figure S1 Pie plots of data filtering and database mapping of the sRNA libraries from *P. nigra* × *deltoids* infected (+rust) and uninfected (-rust) with rust fungi *M. larici-populina*


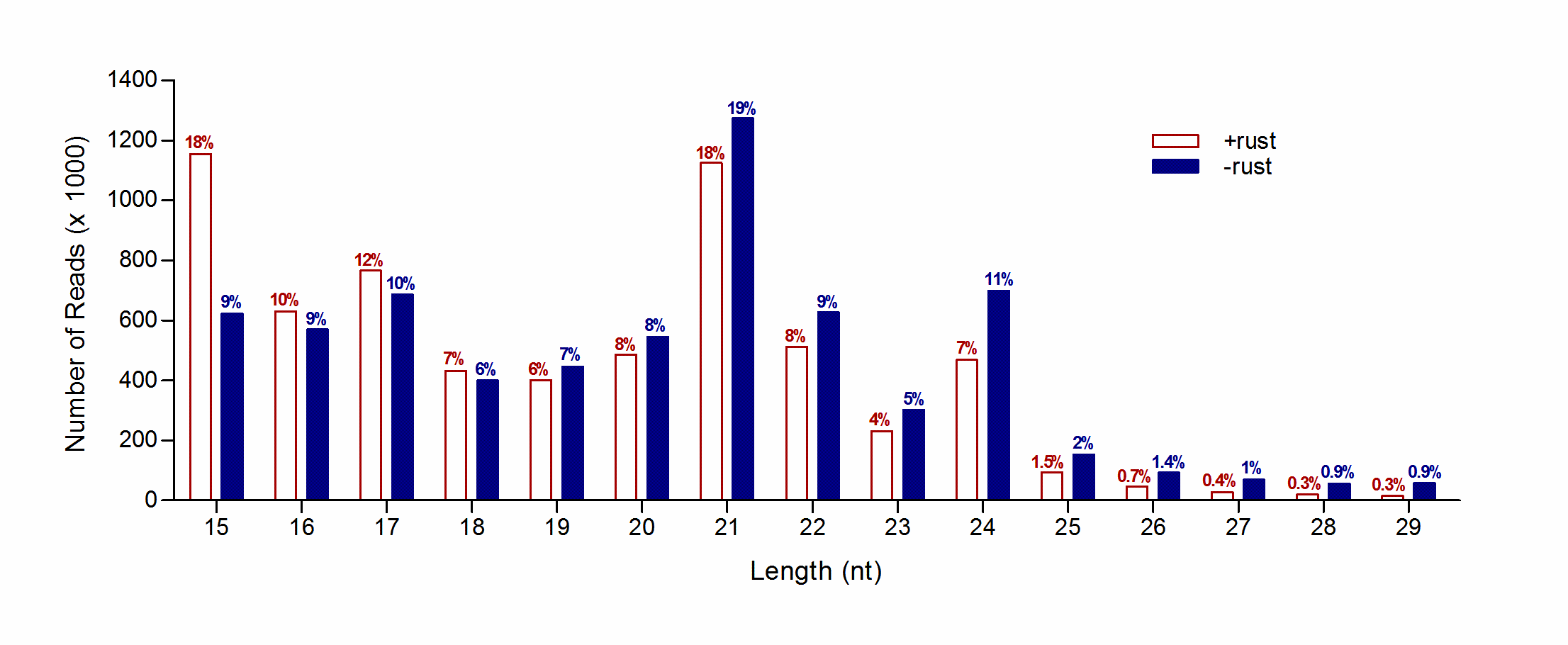


Figure S2 Length distribution of mappable reads in sRNA libraries from *P. nigra* × *deltoids* infected (+rust) and uninfected (-rust) with rust fungi *M. larici-populina*


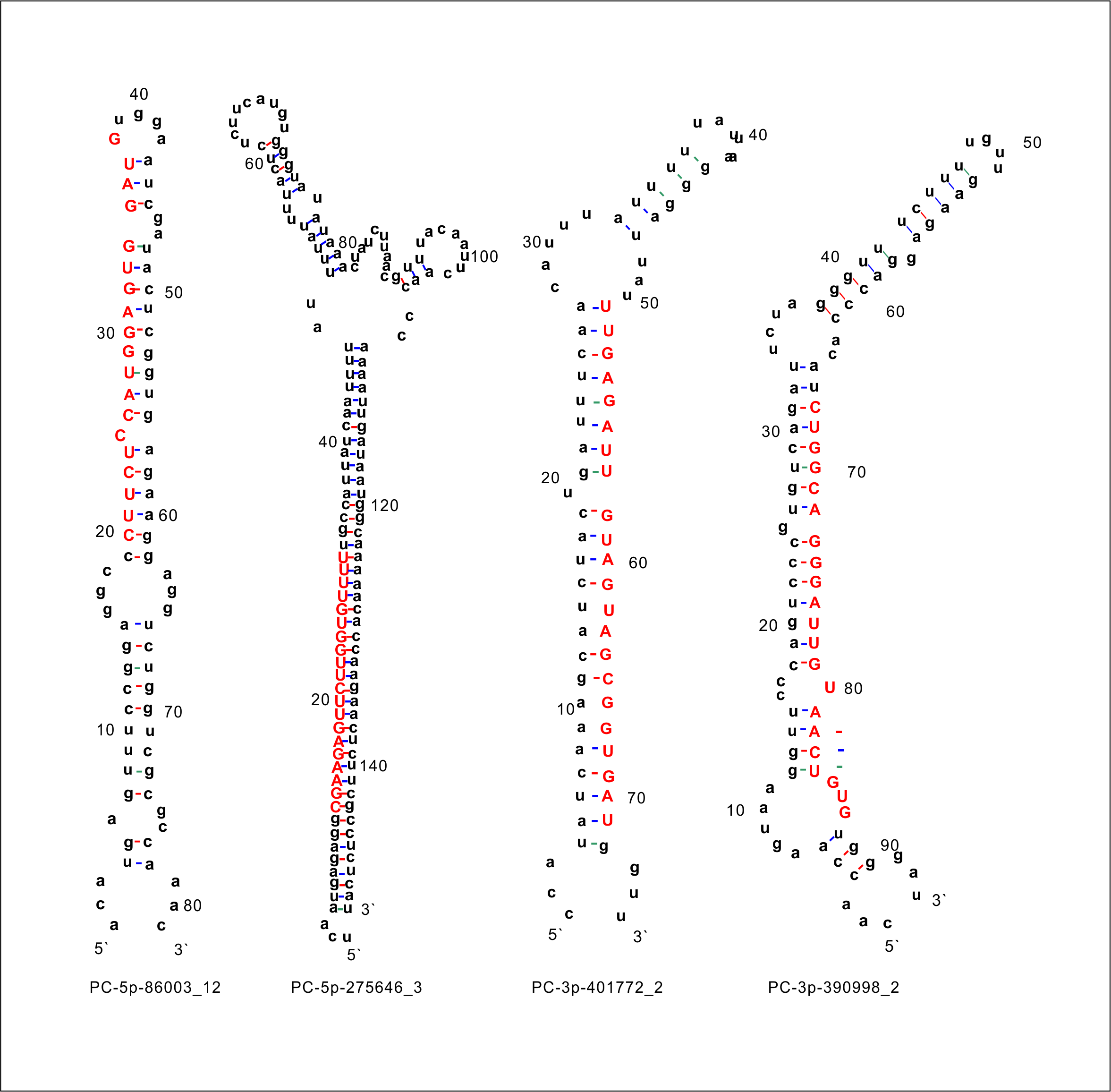


Figure S3 Predicted secondary structures of potential novel miRNAs from *P. nigra* × *deltoids*. Sequences indicated in red correspond to predicted miRNA.


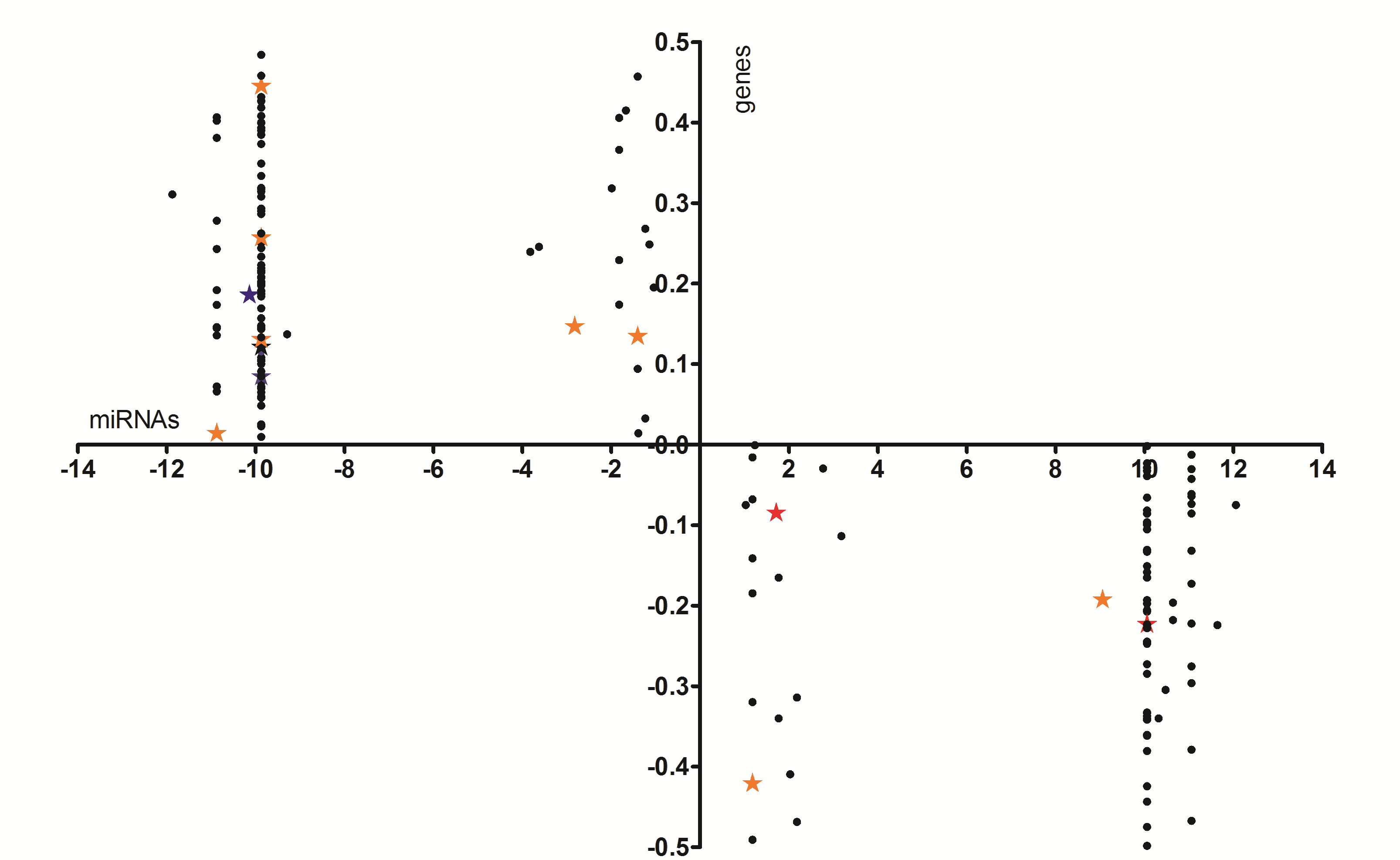


Figure S4 Targets of rust-responsive miRNAs among rust-regulated expressed genes

The x axis is log_2_ (+rust/-rust) transformed fold change of rust-responsive miRNAs, the log_2_ (+rust/-rust) fold changes > 1 are designated as ‘promoted miRNA’, and < −1 are designated as ‘supressed miRNA’. While the y axis stands for un-changed target genes (-0.5< log_2_ fold changes<0.5). The *p*-value is assessed by a 2-tailed chi-square test. The red stars, orange stars and blue stars denote *p*<0.001, 0.001<*p*<0.005 and 0.005<*p*<0.01, respectively.


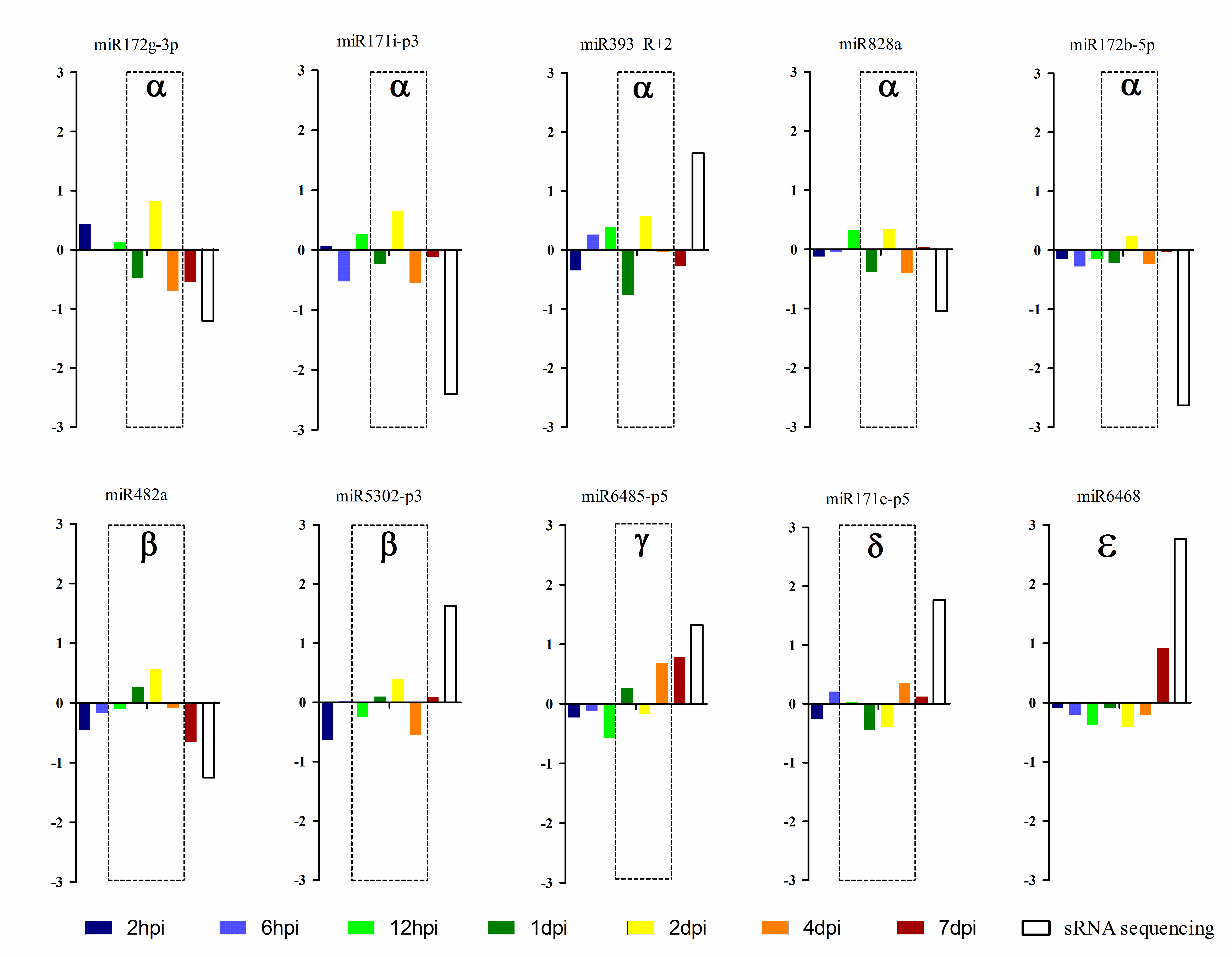


Figure S5 miRNA expression level validated by RT-qPCR at different time points post inoculation


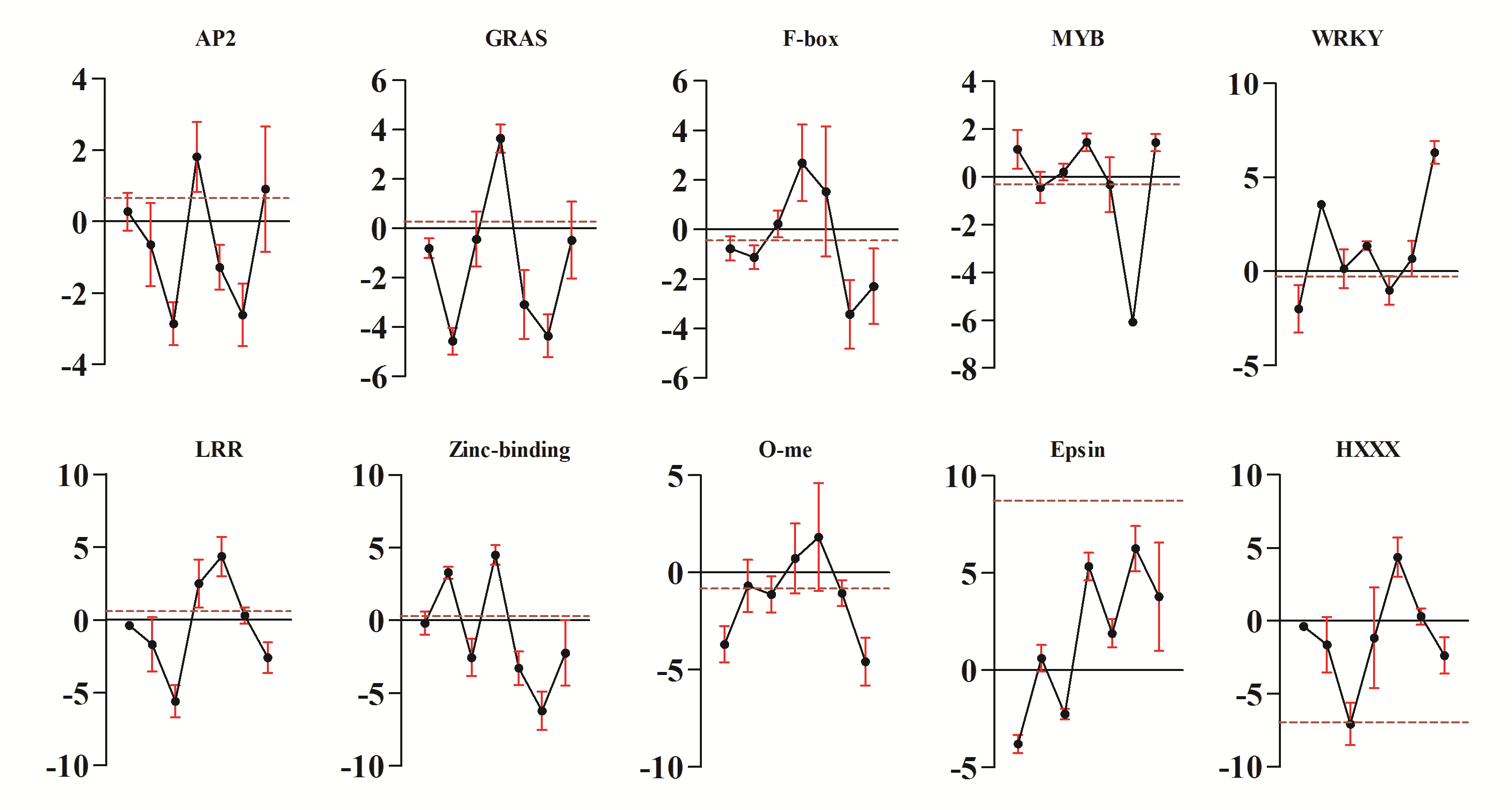


Figure S6 Target gene expression level validated by RT-qPCR at different time points post inoculation

The dashed lines indicated the expression level validated by DGE sequencing of the mixed samples. Error bars indicate SE (n = 3).


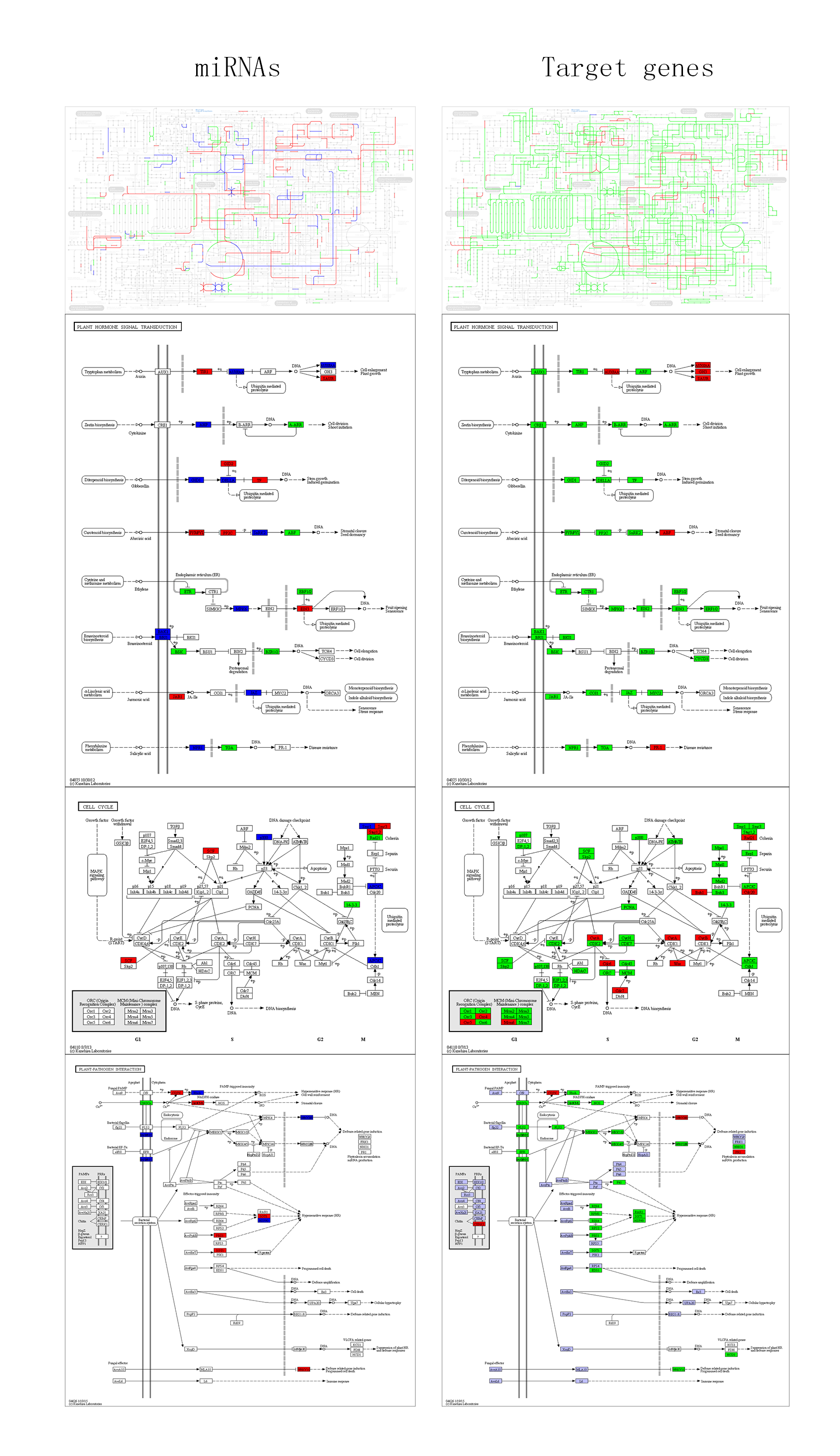


Figure S7 KEGG pathway and atlas

Table S1 A summary of standard data analysis results of the sRNA libraries from *P. nigra* × *deltoids* ‘Robusta’ infected (+rust) and uninfected (-rust) with rust fungi *M. larici-populina*

|  | **Seqseq** | | **%Mappable SequSeq** | | **Unique miRs** | |
| --- | --- | --- | --- | --- | --- | --- |
|  | **+rust** | **-rust** | **+rust** | **-rust** | **+rust** | **-rust** |
| **Total** | 6,420,728 | 6,609,284 | 100% | 100% | 4,070 | 3,820 |
| **Gp 1a** | 636,122 | 750,703 | 9.9% | 11.4% | 438 | 442 |
| **Gp 1b** | 18,510 | 20,164 | 0.3% | 0.3% | 21 | 21 |
| **Gp 2a** | 2,849 | 3,956 | 0% | 0.1% | 76 | 91 |
| **Gp 2b** | 46,822 | 51,152 | 0.7% | 0.8% | 245 | 297 |
| **Gp 3a** | 22,076 | 22,250 | 0.3% | 0.3% | 1,310 | 1,119 |
| **Gp 3b** | 2,261 | 1,988 | 0% | 0% | 1,369 | 1,251 |
| **Gp 4a** | 37,843 | 25,973 | 0.6% | 0.4% | 940 | 920 |
| **Gp 4b** | 1,682,271 | 2,171,567 | 26.2% | 32.9% |  |  |
| **Mapped to other RNAs** | 2,541,815 | 1,963,356 | 39.6% | 29.7% |  |  |
| **Mapped to Repbase** | 141,249 | 120,534 | 2.2% | 1.8% |  |  |
| **Nohit** | 1,393,866 | 1,565,611 | 21.7% | 23.7% |  |  |
| **Raw** | 9,736,262 | 8,840,983 |  |  |  |  |

Table S2 Data summary of degradome library

| **Raw Reads** | **Unique Raw Reads** | **cDNA^a^ Mapped Reads** | **Total Number of input cDNAs** | **Number of coverd cDNAs** |
| --- | --- | --- | --- | --- |
| 8409874 | 3543850 | 5077704 | 73189 | 62858 |

^a^ mRNA database listed in Table 1,B

Table S3 Transcription factors and their regulating miRNA

| **Annotation** | **Log_2_ miRNA** | **Log_2_Target** |
| --- | --- | --- |
| **AP2 family protein** | -1.02 | 0.66 |
| **ARF family protein** | -10.88 | -0.07 |
| **B3 family protein** | -9.88 | 0.05 |
| **BES1 family protein** | 10.06 | -0.48 |
| **bHLH family protein** | 0.18 | -0.01 |
| **bZIP family protein** | 0.86 | -0.12 |
| **C3H family protein** | 10.06 | 0.04 |
| **CO-like family protein** | -0.59 | -0.37 |
| **ERF111** | 12.06 | -0.18 |
| **ERF113** | 1.18 | -0.24 |
| **GRAS family protein** | -2.04 | 0.22 |
| **GRF family protein** | -0.11 | 0.41 |
| **HD-ZIP family protein** | -0.08 | -0.08 |
| **LRR** | -1.25 | 0.50 |
| **MIKC family protein** | 0.86 | -0.12 |
| **MYB** | -1.04 | -0.21573 |
| **NAC family protein** | 0.00 | -0.15 |
| **no apical meristem (NAM) family protein** | 11.06 | 0.01 |
| **SBP family protein** | -0.49 | -0.13 |
| **scarecrow transcription factor family protein** | 0.32 | -0.53 |
| **similar to Auxin response factor 10.** | 0.25 | -0.15 |
| **similar to Auxin response factor 2 (ARF1-binding protein) (ARF1-BP).** | 0.86 | -0.19 |
| **similar to homeobox-leucine zipper protein.** | -9.88 | -1.92 |
| **similar to no apical meristem-like protein.** | -0.38 | 1.16 |
| **similar to PHABULOSA** | -0.65 | 0.58 |
| **similar to SQUAMOSA PROMOTER BINDING PROTEIN-LIKE 7** | -9.88 | -0.42 |
| **TALE family protein** | 1.18 | 0.58 |
| **Trihelix family protein** | -0.23 | 0.05 |
| **zinc finger (C2H2 type) family protein** | -0.14 | 0.17 |

Table S4 Post-transcriptional analyses of miRNA and resistance genes response to the infection of rust in ‘Robusta’

| **Anonomation** | **Regulation**  **models** | **Gene**  **class** | **Target**  **coordinate** | **Log_2_**  **target** | **miRNA** | **miRNA**  **coordinate** | **Log_2_**  **miRNA** |
| --- | --- | --- | --- | --- | --- | --- | --- |
| **MAP kinase 15** | M1a | B1 | 22 | 0.97 | miR1109-p5_1ss7TG | 4 | 10.06 |
| **MAP kinase 15** | M1a | B1 | 23 | 0.96 | miR1109-p5_1ss7TG | 4 | 10.06 |
| **MAP kinase 15** | M1a | B1 | 24 | 0.96 | miR1109-p5_1ss7TG | 4 | 10.06 |
| **MAP kinase 15** | M1a | B1 | 25 | 0.96 | miR1109-p5_1ss7TG | 4 | 10.06 |
| **MAP kinase 15** | M1a | B1 | 26 | 0.96 | miR1109-p5_1ss7TG | 4 | 10.06 |
| **MAP kinase 15** | M1a | B1 | 27 | 0.95 | miR1109-p5_1ss7TG | 4 | 10.06 |
| **MAP kinase 15** | M1a | B1 | 28 | 0.95 | miR1109-p5_1ss7TG | 4 | 10.06 |
| **MAP kinase 15** | M1a | B1 | 29 | 0.94 | miR1109-p5_1ss7TG | 4 | 10.06 |
| **Glutaredoxin family protein** | M1a | B6 | 49 | 0.90 | miR164-p3 | 25 | 1.18 |
| **WRKY DNA-binding protein 11** | M1a | C1 | 55 | 0.79 | miR2097-p3 | 7 | 10.06 |
| **WRKY DNA-binding protein 11** | M1a | C1 | 56 | 0.79 | miR2097-p3 | 7 | 10.06 |
| **WRKY DNA-binding protein 2** | M1a | C1 | 57 | 0.78 | miR2097-p3 | 7 | 10.06 |
| **Disease resistance protein (TIR-NBS-LRR class) family** | M1b | A2 | 6 | 0.49 | miR482 | 18 | 1.77 |
| **F-box/RNI-like superfamily protein** | M1b | E3 | 164 | 0.43 | PC-5p-3009831_1 | 2 | 11.06 |
| **F-box/RNI-like superfamily protein** | M1b | E3 | 164 | 0.43 | miR393a_R+2 | 20 | 1.64 |
| **Basic-leucine zipper (bZIP) transcription factor family protein** | M1b | C3 | 93 | 0.36 | miR1082a-p5 | 10 | 10.06 |
| **Disease resistance protein (TIR-NBS-LRR class) family** | M1b | A2 | 7 | 0.35 | miR482 | 18 | 1.77 |
| **Basic-leucine zipper (bZIP) transcription factor family protein** | M1b | C3 | 92 | 0.33 | miR1082a-p5 | 10 | 10.06 |
| **WRKY DNA-binding protein 2** | M1b | C1 | 59 | 0.31 | miR156-p3 | 8 | 10.06 |
| **WRKY family transcription factor** | M1b | C2 | 81 | 0.29 | miR394-p3 | 9 | 10.06 |
| **WRKY family transcription factor family protein** | M1b | C2 | 82 | 0.28 | miR394-p3 | 9 | 10.06 |
| **WRKY family transcription factor family protein** | M1b | C2 | 83 | 0.28 | miR394-p3 | 9 | 10.06 |
| **WRKY family transcription factor family protein** | M1b | C2 | 84 | 0.27 | miR394-p3 | 9 | 10.06 |
| **WRKY family transcription factor family protein** | M1b | C2 | 85 | 0.27 | miR394-p3 | 9 | 10.06 |
| **myb-like HTH transcriptional regulator family protein** | M1b | C6 | 112 | 0.26 | miR166m-p5 | 24 | 1.18 |
| **WRKY family transcription factor family protein** | M1b | C2 | 86 | 0.25 | miR394-p3 | 9 | 10.06 |
| **myb-like HTH transcriptional regulator family protein** | M1b | C6 | 113 | 0.25 | miR166m-p5 | 24 | 1.18 |
| **myb-like HTH transcriptional regulator family protein** | M1b | C6 | 114 | 0.25 | miR166m-p5 | 24 | 1.18 |
| **WRKY family transcription factor family protein** | M1b | C2 | 87 | 0.24 | miR394-p3 | 9 | 10.06 |
| **myb-like HTH transcriptional regulator family protein** | M1b | C6 | 115 | 0.24 | miR166m-p5 | 24 | 1.18 |
| **myb-like HTH transcriptional regulator family protein** | M1b | C6 | 110 | 0.24 | miR166m-p5 | 24 | 1.18 |
| **Disease resistance protein (TIR-NBS-LRR class) family** | M1b | A2 | 9 | 0.23 | miR482 | 18 | 1.77 |
| **myb-like HTH transcriptional regulator family protein** | M1b | C6 | 116 | 0.22 | miR166m-p5 | 24 | 1.18 |
| **WRKY family transcription factor family protein** | M1b | C2 | 88 | 0.21 | miR394-p3 | 9 | 10.06 |
| **WRKY family transcription factor family protein** | M1b | C2 | 89 | 0.21 | miR394-p3 | 9 | 10.06 |
| **myb-like HTH transcriptional regulator family protein** | M1b | C6 | 117 | 0.21 | miR166m-p5 | 24 | 1.18 |
| **myb-like HTH transcriptional regulator family protein** | M1b | C6 | 111 | 0.20 | miR166m-p5 | 24 | 1.18 |
| **WRKY DNA-binding protein 2** | M1b | C1 | 61 | 0.16 | miR394-p3 | 9 | 10.06 |
| **Disease resistance protein (TIR-NBS-LRR class) family** | M1b | A2 | 10 | 0.16 | miR482 | 18 | 1.77 |
| **disease resistance protein (TIR-NBS-LRR class), putative** | M1b | A2 | 11 | 0.12 | miR482 | 18 | 1.77 |
| **WRKY DNA-binding protein 23** | M1b | C1 | 62 | 0.11 | miR394-p3 | 9 | 10.06 |
| **Pathogenesis-related thaumatin superfamily protein** | M1b | E6 | 176 | 0.10 | miR1082a-p5 | 10 | 10.06 |
| **peptidoglycan-binding LysM domain-containing protein** | M1b | E7 | 178 | 0.07 | PC-5p-693124_1 | 16 | 10.06 |
| **WRKY DNA-binding protein 28** | M1b | C1 | 64 | 0.05 | miR394-p3 | 9 | 10.06 |
| **disease resistance protein (TIR-NBS-LRR class), putative** | M1b | A2 | 12 | 0.04 | miR482 | 18 | 1.77 |
| **ascorbate peroxidase 2** | M1b | D3 | 128 | 0.03 | #N/A | 12 | 10.06 |
| **WRKY DNA-binding protein 3** | M1b | C1 | 66 | 0.01 | miR156-p3 | 8 | 10.06 |
| **manganese superoxide dismutase 1** | M1b | D2 | 127 | 0.01 | PC-5p-1476828_1 | 11 | 10.06 |
| **WRKY DNA-binding protein 3** | M1b | C1 | 67 | 0.00 | miR394-p3 | 9 | 10.06 |
| **disease resistance protein (TIR-NBS-LRR class), putative** | M1b | A2 | 13 | -0.05 | miR482 | 18 | 1.77 |
| **phospholipase D alpha 1** | M1b | E8 | 182 | -0.07 | miR4233-p5 | 19 | 1.77 |
| **disease resistance protein (TIR-NBS-LRR class), putative** | M1b | A2 | 15 | -0.09 | miR482 | 18 | 1.77 |
| **glutathione S-transferase F11** | M1b | D7 | 144 | -0.11 | miR319g-p5_1ss7AC | 13 | 10.06 |
| **phospholipase D alpha 1** | M1b | E8 | 181 | -0.11 | miR4233-p5 | 19 | 1.77 |
| **glutathione reductase** | M1b | D6 | 140 | -0.12 | miR395x-p3 | 1 | 11.64 |
| **peptidoglycan-binding LysM domain-containing protein** | M1b | E7 | 179 | -0.13 | PC-5p-693124_1 | 16 | 10.06 |
| **WRKY DNA-binding protein 32** | M1b | C1 | 72 | -0.17 | miR156-p3 | 8 | 10.06 |
| **F-box/RNI-like superfamily protein** | M1b | E3 | 165 | -0.23 | PC-5p-3009831_1 | 2 | 11.06 |
| **F-box/RNI-like superfamily protein** | M1b | E3 | 165 | -0.23 | miR393_R+2 | 20 | 1.64 |
| **pathogenesis related homeodomain protein A** | M1b | E4 | 168 | -0.28 | miR3447 | 3 | 11.06 |
| **pathogenesis related homeodomain protein A** | M1b | E4 | 169 | -0.28 | miR3447 | 3 | 11.06 |
| **pathogenesis related homeodomain protein A** | M1b | E4 | 167 | -0.34 | miR3447 | 3 | 11.06 |
| **pathogenesis related homeodomain protein A** | M1b | E4 | 170 | -0.34 | miR3447 | 3 | 11.06 |
| **Glutaredoxin family protein** | M1b | B6 | 50 | -0.39 | miR1027a-p5 | 6 | 10.06 |
| **WRKY DNA-binding protein 75** | M1b | C1 | 79 | -0.39 | miR858a-p3 | 5 | 10.06 |
| **pathogenesis related homeodomain protein A** | M1b | E4 | 171 | -0.45 | miR3447 | 3 | 11.06 |
| **Glutaredoxin family protein** | M1b | B6 | 51 | -0.45 | miR1027a-p5 | 6 | 10.06 |
| **F-box family protein** | M1b | E2 | 160 | -0.46 | PC-3p-1442548_1 | 14 | 10.06 |
| **Wound-responsive family protein** | M1b | E9 | 190 | -0.47 | miR164a-p3 | 21 | 1.44 |
| **ferritin 2** | M1b | D8 | 149 | -0.47 | PC-5p-86003_12 | 26 | 1.18 |
| **F-box family protein** | M1b | E2 | 161 | -0.48 | PC-3p-1442548_1 | 14 | 10.06 |
| **ferritin 2** | M1b | D8 | 148 | -0.49 | miR164-p3 | 25 | 1.18 |
| **ferritin 2** | M1b | D8 | 148 | -0.49 | PC-5p-86003_12 | 26 | 1.18 |
| **ethylene responsive element binding factor 1** | M1c | C4 | 100 | -0.53 | miR164-p3 | 25 | 1.18 |
| **F-box family protein** | M1c | E2 | 162 | -0.65 | miR394-p3 | 9 | 10.06 |
| **Kunitz family trypsin and protease inhibitor protein** | M1c | B4 | 39 | -0.68 | miR319i-p3 | 22 | 1.18 |
| **Kunitz family trypsin and protease inhibitor protein** | M1c | B4 | 41 | -0.71 | miR319i-p3 | 22 | 1.18 |
| **Kunitz family trypsin and protease inhibitor protein** | M1c | B4 | 40 | -0.72 | miR319i-p3 | 22 | 1.18 |
| **MAP kinase kinase 9** | M1c | B1 | 33 | -0.78 | miR858a-p3 | 5 | 10.06 |
| **ethylene responsive element binding factor 1** | M1c | C4 | 101 | -0.78 | miR1109-p5_1ss7TG | 4 | 10.06 |
| **Wound-responsive family protein** | M1c | E9 | 191 | -0.99 | miR6443-p5 | 17 | 10.06 |
| **Wound-responsive family protein** | M1c | E9 | 188 | -1.00 | miR6443-p5 | 17 | 10.06 |
| **Disease resistance protein (TIR-NBS-LRR class) family** | M1c | A2 | 20 | -1.00 | miR482 | 18 | 1.77 |
| **pathogenesis-related 4** | M1c | E5 | 173 | -1.02 | miR2864 | 15 | 10.06 |
| **pathogenesis-related 4** | M1c | E5 | 173 | -1.02 | miR5517-p5 | 23 | 1.18 |
| **Wound-responsive family protein** | M1c | E9 | 192 | -1.10 | miR164a-p3 | 21 | 1.44 |
| **Wound-responsive family protein** | M1c | E9 | 193 | -1.12 | miR6443-p5 | 17 | 10.06 |
| **Wound-responsive family protein** | M1c | E9 | 194 | -1.23 | miR394-p3 | 9 | 10.06 |
| **Wound-responsive family protein** | M2a | E9 | 189 | 1.39 | miR5293-p5 | 40 | -0.18 |
| **Glutaredoxin family protein** | M2a | B6 | 49 | 0.90 | miR2606b-p3 | 35 | 0.18 |
| **Glutaredoxin family protein** | M2a | B6 | 48 | 0.58 | miR6300 | 44 | -0.82 |
| **Disease resistance protein (TIR-NBS-LRR class) family** | M2a | A2 | 5 | 0.50 | miR472b | 41 | -0.22 |
| **F-box/RNI-like superfamily protein** | M2b | E3 | 164 | 0.43 | miR393a-5p | 28 | 0.68 |
| **F-box/RNI-like superfamily protein** | M2b | E3 | 164 | 0.43 | miR393a-5p_R+1 | 29 | 0.52 |
| **F-box/RNI-like superfamily protein** | M2b | E3 | 164 | 0.43 | miR393a_R+1 | 30 | 0.50 |
| **Disease resistance protein (TIR-NBS-LRR class) family** | M2b | A2 | 4 | 0.37 | miR472b | 41 | -0.22 |
| **Disease resistance protein (TIR-NBS-LRR class) family** | M2b | A2 | 8 | 0.30 | miR472b | 41 | -0.22 |
| **Disease resistance protein (CC-NBS-LRR class) family** | M2b | A1 | 1 | 0.21 | miR482b | 38 | 0.02 |
| **Pathogenesis-related thaumatin superfamily protein** | M2b | E6 | 175 | 0.14 | miR1509 | 49 | -0.99 |
| **Pathogenesis-related thaumatin superfamily protein** | M2b | E6 | 176 | 0.10 | miR408 | 27 | 0.77 |
| **Pathogenesis-related thaumatin superfamily protein** | M2b | E6 | 176 | 0.10 | miR1509 | 49 | -0.99 |
| **F-box family protein** | M2b | E2 | 154 | 0.04 | miR827a-p5 | 33 | 0.18 |
| **F-box family protein** | M2b | E2 | 155 | 0.02 | miR827a-p5 | 33 | 0.18 |
| **F-box family protein** | M2b | E2 | 153 | 0.02 | miR6424-p3 | 36 | 0.05 |
| **F-box family protein** | M2b | E2 | 153 | 0.02 | miR159d-p3 | 37 | 0.03 |
| **WRKY family transcription factor family protein** | M2b | C2 | 90 | 0.01 | miR5542-p3 | 47 | -0.82 |
| **disease resistance protein (TIR-NBS-LRR class), putative** | M2b | A2 | 13 | -0.05 | miR482c | 42 | -0.35 |
| **F-box family protein** | M2b | E2 | 156 | -0.06 | miR827a-p5 | 33 | 0.18 |
| **F-box family protein** | M2b | E2 | 157 | -0.06 | miR827a-p5 | 33 | 0.18 |
| **WRKY DNA-binding protein 32** | M2b | C1 | 69 | -0.07 | PC-3p-152457_6 | 46 | -0.82 |
| **WRKY DNA-binding protein 32** | M2b | C1 | 70 | -0.07 | PC-3p-152457_6 | 46 | -0.82 |
| **disease resistance protein (TIR-NBS-LRR class), putative** | M2b | A2 | 15 | -0.09 | miR482c | 42 | -0.35 |
| **F-box and associated interaction domains-containing protein** | M2b | E1 | 151 | -0.18 | miR2096-p5 | 48 | -0.82 |
| **F-box/RNI-like superfamily protein** | M2b | E3 | 165 | -0.23 | miR393a-5p | 28 | 0.68 |
| **F-box/RNI-like superfamily protein** | M2b | E3 | 165 | -0.23 | miR393a-5p_R+1 | 29 | 0.52 |
| **F-box/RNI-like superfamily protein** | M2b | E3 | 165 | -0.23 | miR393a_R+1 | 30 | 0.50 |
| **MAP kinase 20** | M2b | B1 | 31 | -0.23 | miR2661-p5 | 34 | 0.18 |
| **MAP kinase 6** | M2b | B1 | 32 | -0.24 | miR2661-p5 | 34 | 0.18 |
| **ascorbate peroxidase 2** | M2b | D3 | 129 | -0.25 | miR1511_R-3 | 32 | 0.18 |
| **ascorbate peroxidase 2** | M2b | D3 | 130 | -0.26 | miR1511_R-3 | 32 | 0.18 |
| **ascorbate peroxidase 2** | M2b | D3 | 131 | -0.26 | miR1511_R-3 | 32 | 0.18 |
| **ascorbate peroxidase 3** | M2b | D3 | 132 | -0.27 | miR1511_R-3 | 32 | 0.18 |
| **Disease resistance protein (CC-NBS-LRR class) family** | M2b | A1 | 2 | -0.30 | miR482b | 38 | 0.02 |
| **ethylene responsive element binding factor 1** | M2c | C4 | 100 | -0.53 | PC-5p-80622_13 | 31 | 0.40 |
| **bZIP transcription factor family protein** | M2c | C3 | 97 | -0.87 | miR5826-p5 | 45 | -0.82 |
| **Disease resistance protein (TIR-NBS-LRR class) family** | M2c | A2 | 20 | -1.00 | miR472a | 39 | -0.17 |
| **Disease resistance protein (TIR-NBS-LRR class) family** | M2c | A2 | 20 | -1.00 | miR472b | 41 | -0.22 |
| **Wound-responsive family protein** | M2c | E9 | 195 | -1.25 | miR171a-p3 | 43 | -0.36 |
| **WRKY DNA-binding protein 11** | M3a | C1 | 53 | 1.00 | PC-3p-335670_2 | 80 | -10.88 |
| **WRKY DNA-binding protein 11** | M3a | C1 | 54 | 1.00 | PC-3p-335670_2 | 80 | -10.88 |
| **WRKY DNA-binding protein 11** | M3a | C1 | 55 | 0.79 | miR6426b-p5 | 58 | -9.88 |
| **WRKY DNA-binding protein 11** | M3a | C1 | 56 | 0.79 | miR6426b-p5 | 58 | -9.88 |
| **WRKY DNA-binding protein 2** | M3a | C1 | 57 | 0.78 | miR6426b-p5 | 58 | -9.88 |
| **WRKY DNA-binding protein 2** | M3a | C1 | 58 | 0.63 | PC-3p-335670_2 | 80 | -10.88 |
| **glutathione S-transferase 6** | M3a | D7 | 142 | 0.54 | PC-3p-1909651_1 | 71 | -9.88 |
| **Disease resistance protein (TIR-NBS-LRR class) family** | M3a | A2 | 5 | 0.50 | miR482a | 50 | -1.25 |
| **Disease resistance protein (TIR-NBS-LRR class) family** | M3a | A2 | 5 | 0.50 | miR482d | 76 | -10.88 |
| **F-box/RNI-like superfamily protein** | M3b | E3 | 164 | 0.43 | PC-3p-2026321_1 | 74 | -9.88 |
| **glutathione S-transferase F11** | M3b | D7 | 143 | 0.40 | miR4354-p5 | 72 | -9.88 |
| **Disease resistance protein (TIR-NBS-LRR class) family** | M3b | A2 | 4 | 0.37 | miR482a2 | 50 | -1.25 |
| **monodehydroascorbate reductase 4** | M3b | D4 | 134 | 0.37 | miR2608-p3 | 54 | -2.49 |
| **Disease resistance protein (TIR-NBS-LRR class) family** | M3b | A2 | 4 | 0.37 | miR482d | 76 | -10.88 |
| **Disease resistance protein (TIR-NBS-LRR class) family** | M3b | A2 | 8 | 0.30 | miR482a2 | 50 | -1.25 |
| **Disease resistance protein (TIR-NBS-LRR class) family** | M3b | A2 | 8 | 0.30 | miR482d | 76 | -10.88 |
| **WRKY family transcription factor family protein** | M3b | C2 | 84 | 0.27 | miR437v-p5 | 62 | -9.88 |
| **WRKY family transcription factor family protein** | M3b | C2 | 86 | 0.25 | miR437v-p5 | 62 | -9.88 |
| **MAP kinase 20** | M3b | B1 | 30 | 0.23 | miR399d-p5 | 78 | -10.88 |
| **WRKY family transcription factor family protein** | M3b | C2 | 89 | 0.21 | miR437v-p5 | 62 | -9.88 |
| **WRKY DNA-binding protein 2** | M3b | C1 | 60 | 0.16 | miR394-p5 | 59 | -9.88 |
| **phospholipase D alpha 1** | M3b | E8 | 186 | 0.08 | miR530b-p3 | 75 | -9.88 |
| **phospholipase D alpha 1** | M3b | E8 | 185 | 0.08 | miR530b-p3 | 75 | -9.88 |
| **phospholipase D alpha 1** | M3b | E8 | 183 | 0.07 | miR530b-p3 | 75 | -9.88 |
| **WRKY DNA-binding protein 26** | M3b | C1 | 63 | 0.06 | PC-3p-123255_8 | 51 | -1.40 |
| **phospholipase D alpha 1** | M3b | E8 | 184 | 0.06 | miR530b-p3 | 75 | -9.88 |
| **WRKY DNA-binding protein 26** | M3b | C1 | 63 | 0.06 | miR172f-p5 | 60 | -9.88 |
| **2-cysteine peroxiredoxin B** | M3b | B5 | 43 | 0.03 | PC-5p-897300_1 | 57 | -9.88 |
| **2-cysteine peroxiredoxin B** | M3b | B5 | 44 | 0.03 | PC-5p-897300_1 | 57 | -9.88 |
| **2-cysteine peroxiredoxin B** | M3b | B5 | 45 | 0.03 | PC-5p-897300_1 | 57 | -9.88 |
| **Basic-leucine zipper (bZIP) transcription factor family protein** | M3b | C3 | 94 | 0.03 | miR6426b-p5 | 58 | -9.88 |
| **WRKY DNA-binding protein 28** | M3b | C1 | 65 | 0.02 | PC-3p-123255_8 | 51 | -1.40 |
| **WRKY DNA-binding protein 28** | M3b | C1 | 65 | 0.02 | miR172f-p5 | 60 | -9.88 |
| **Basic-leucine zipper (bZIP) transcription factor family protein** | M3b | C3 | 95 | 0.00 | miR6426b-p5 | 58 | -9.88 |
| **ethylene responsive element binding factor 1** | M3b | C4 | 99 | 0.00 | miR395d-p5 | 61 | -9.88 |
| **2-cysteine peroxiredoxin B** | M3b | B5 | 46 | -0.01 | PC-5p-897300_1 | 57 | -9.88 |
| **disease resistance protein (TIR-NBS-LRR class), putative** | M3b | A2 | 14 | -0.06 | miR414-p5 | 52 | -1.40 |
| **WRKY DNA-binding protein 3** | M3b | C1 | 68 | -0.06 | miR172f-p5 | 60 | -9.88 |
| **disease resistance protein (TIR-NBS-LRR class), putative** | M3b | A2 | 14 | -0.06 | miR845-p3 | 77 | -10.88 |
| **F-box family protein** | M3b | E2 | 158 | -0.09 | miR157d-p3 | 73 | -9.88 |
| **MAPK/ERK kinase kinase 3** | M3b | B2 | 35 | -0.10 | PC-3p-1943411_1 | 55 | -9.88 |
| **Basic-leucine zipper (bZIP) transcription factor family protein** | M3b | C3 | 96 | -0.10 | miR6426b-p5 | 58 | -9.88 |
| **Disease resistance protein (TIR-NBS-LRR class) family** | M3b | A2 | 16 | -0.12 | miR414-p5 | 52 | -1.40 |
| **Disease resistance protein (TIR-NBS-LRR class) family** | M3b | A2 | 16 | -0.12 | miR845-p3 | 77 | -10.88 |
| **WRKY DNA-binding protein 32** | M3b | C1 | 71 | -0.15 | miR395d-p5 | 61 | -9.88 |
| **glutathione peroxidase 4** | M3b | D5 | 137 | -0.15 | miR160-p3 | 70 | -9.88 |
| **glutathione peroxidase 4** | M3b | D5 | 138 | -0.16 | miR160-p3 | 70 | -9.88 |
| **MAPK/ERK kinase kinase 3** | M3b | B2 | 36 | -0.18 | PC-3p-1943411_1 | 55 | -9.88 |
| **WRKY DNA-binding protein 32** | M3b | C1 | 73 | -0.18 | miR172f-p5 | 60 | -9.88 |
| **copper/zinc superoxide dismutase 2** | M3b | D1 | 122 | -0.18 | miR6250-p5 | 68 | -9.88 |
| **Disease resistance protein (TIR-NBS-LRR class) family** | M3b | A2 | 17 | -0.19 | miR414-p5 | 52 | -1.40 |
| **Disease resistance protein (TIR-NBS-LRR class) family** | M3b | A2 | 17 | -0.19 | miR845-p3 | 77 | -10.88 |
| **glutathione S-transferase THETA 2** | M3b | D7 | 145 | -0.19 | miR164c-p5 | 81 | -10.88 |
| **WRKY DNA-binding protein 32** | M3b | C1 | 74 | -0.20 | miR395d-p5 | 61 | -9.88 |
| **Glutathione S-transferase, C-terminal-like;Translation elongation factor EF1B/ribosomal protein S6** | M3b | D7 | 146 | -0.20 | miR6250-p5 | 68 | -9.88 |
| **copper/zinc superoxide dismutase 2** | M3b | D1 | 123 | -0.20 | miR6250-p5 | 68 | -9.88 |
| **copper/zinc superoxide dismutase 2** | M3b | D1 | 124 | -0.20 | miR6250-p5 | 68 | -9.88 |
| **copper/zinc superoxide dismutase 2** | M3b | D1 | 125 | -0.21 | miR6250-p5 | 68 | -9.88 |
| **WRKY DNA-binding protein 40** | M3b | C1 | 75 | -0.21 | miR395d-p5 | 61 | -9.88 |
| **jasmonate-zim-domain protein 3** | M3b | C7 | 119 | -0.21 | miR396d-p5 | 67 | -9.88 |
| **F-box family protein** | M3b | E2 | 159 | -0.23 | miR157d-p3 | 73 | -9.88 |
| **WRKY DNA-binding protein 7** | M3b | C1 | 76 | -0.23 | miR395d-p5 | 61 | -9.88 |
| **WRKY DNA-binding protein 7** | M3b | C1 | 77 | -0.23 | miR395d-p5 | 61 | -9.88 |
| **WRKY DNA-binding protein 7** | M3b | C1 | 78 | -0.23 | miR395d-p5 | 61 | -9.88 |
| **MAPK/ERK kinase kinase 3** | M3b | B2 | 37 | -0.26 | PC-3p-1943411_1 | 55 | -9.88 |
| **Disease resistance protein (TIR-NBS-LRR class) family** | M3b | A2 | 18 | -0.26 | miR845-p3 | 77 | -10.88 |
| **Disease resistance protein (TIR-NBS-LRR class) family** | M3b | A2 | 19 | -0.30 | miR845-p3 | 77 | -10.88 |
| **jasmonate-zim-domain protein 3** | M3b | C7 | 120 | -0.31 | miR396d-p5 | 67 | -9.88 |
| **wound-responsive protein-related** | M3b | E10 | 199 | -0.36 | PC-5p-435547_2 | 82 | -10.88 |
| **glutathione peroxidase 1** | M3b | D5 | 136 | -0.40 | miR536a-p5 | 69 | -9.88 |
| **wound-responsive protein-related** | M3b | E10 | 197 | -0.44 | miR393b-p5 | 65 | -9.88 |
| **wound-responsive protein-related** | M3b | E10 | 201 | -0.44 | miR393b-p5 | 65 | -9.88 |
| **wound-responsive protein-related** | M3b | E10 | 198 | -0.44 | miR393b-p5 | 65 | -9.88 |
| **wound-responsive protein-related** | M3b | E10 | 200 | -0.44 | PC-5p-435547_2 | 82 | -10.88 |
| **wound-responsive protein-related** | M3b | E10 | 197 | -0.44 | PC-5p-435547_2 | 82 | -10.88 |
| **wound-responsive protein-related** | M3b | E10 | 201 | -0.44 | PC-5p-435547_2 | 82 | -10.88 |
| **wound-responsive protein-related** | M3b | E10 | 198 | -0.44 | PC-5p-435547_2 | 82 | -10.88 |
| **myb family transcription factor** | M3b | C5 | 103 | -0.45 | miR6426b-p5 | 58 | -9.88 |
| **Wound-responsive family protein** | M3b | E9 | 190 | -0.47 | miR393b-p5 | 65 | -9.88 |
| **myb family transcription factor** | M3b | C5 | 104 | -0.48 | miR6426b-p5 | 58 | -9.88 |
| **myb family transcription factor** | M3b | C5 | 104 | -0.48 | miR393b-p5 | 65 | -9.88 |
| **myb family transcription factor** | M3b | C5 | 105 | -0.67 | miR6426b-p5 | 58 | -9.88 |
| **myb family transcription factor** | M3c | C5 | 105 | -0.67 | miR6426b-p5 | 58 | -9.88 |
| **Kunitz family trypsin and protease inhibitor protein** | M3c | B4 | 39 | -0.68 | miR168-p5 | 56 | -9.88 |
| **myb family transcription factor** | M3c | C5 | 106 | -0.69 | miR6426b-p5 | 58 | -9.88 |
| **myb family transcription factor** | M3c | C5 | 106 | -0.69 | miR6426b-p5 | 58 | -9.88 |
| **myb family transcription factor** | M3c | C5 | 106 | -0.69 | miR172-p5 | 66 | -9.88 |
| **Kunitz family trypsin and protease inhibitor protein** | M3c | B4 | 41 | -0.71 | miR168-p5 | 56 | -9.88 |
| **Kunitz family trypsin and protease inhibitor protein** | M3c | B4 | 40 | -0.72 | miR168-p5 | 56 | -9.88 |
| **ethylene responsive element binding factor 1** | M3c | C4 | 101 | -0.78 | miR845-p5 | 64 | -9.88 |
| **MAP kinase kinase 9** | M3c | B1 | 33 | -0.78 | miR443-p3 | 79 | -10.88 |
| **myb family transcription factor** | M3c | C5 | 107 | -0.79 | miR6426b-p5 | 58 | -9.88 |
| **myb family transcription factor** | M3c | C5 | 107 | -0.79 | miR6426b-p5 | 58 | -9.88 |
| **myb family transcription factor** | M3c | C5 | 107 | -0.79 | miR172-p5 | 66 | -9.88 |
| **myb family transcription factor** | M3c | C5 | 108 | -0.80 | miR6426b-p5 | 58 | -9.88 |
| **myb family transcription factor** | M3c | C5 | 108 | -0.80 | miR6426b-p5 | 58 | -9.88 |
| **myb family transcription factor** | M3c | C5 | 108 | -0.80 | miR172-p5 | 66 | -9.88 |
| **bZIP transcription factor family protein** | M3c | C3 | 97 | -0.87 | miR167a-p3 | 63 | -9.88 |
| **Disease resistance protein (TIR-NBS-LRR class) family** | M3c | A2 | 20 | -1.00 | miR5301-p3 | 53 | -1.67 |

Table S5 The Gene Ontology of the rust-responsive gene and their regulating miRNA

| **No.** | **log_2_FoldChange** | **pval** | **FDR** | **GO_Term** |
| --- | --- | --- | --- | --- |
| 1 | 17.17 | 0.00 | 0.02 |  |
| 2 | 17.17 | 0.00 | 0.00 | GO:0055085(transmembrane transport) |
| 3 | 17.17 | 0.00 | 0.00 |  |
| 4 | 5.51 | 0.00 | 0.00 |  |
| 5 | 5.28 | 0.00 | 0.00 | GO:0020037(heme binding),GO:0006979(response to oxidative stress) |
| 6 | 5.13 | 0.00 | 0.00 |  |
| 7 | 4.96 | 0.00 | 0.00 |  |
| 8 | 4.81 | 0.00 | 0.00 |  |
| 9 | 4.66 | 0.00 | 0.00 |  |
| 10 | 4.13 | 0.00 | 0.03 |  |
| 11 | 4.13 | 0.00 | 0.03 |  |
| 12 | 4.13 | 0.00 | 0.03 |  |
| 13 | 4.13 | 0.00 | 0.03 | GO:0006857(oligopeptide transport),GO:0016020(membrane),GO:0055085(transmembrane transport),GO:0006857(oligopeptide transport),GO:0016020(membrane),GO:0055085(transmembrane transport) |
| 14 | 4.13 | 0.00 | 0.03 | GO:0006857(oligopeptide transport),GO:0016020(membrane),GO:0055085(transmembrane transport),GO:0006857(oligopeptide transport),GO:0016020(membrane),GO:0055085(transmembrane transport) |
| 15 | 4.04 | 0.00 | 0.05 | GO:0005543(phospholipid binding) |
| 16 | 3.42 | 0.00 | 0.02 |  |
| 17 | 3.42 | 0.00 | 0.00 | GO:0016491(oxidoreductase activity),GO:0050660(flavin adenine dinucleotide binding),GO:0016491(oxidoreductase activity),GO:0050660(flavin adenine dinucleotide binding) |
| 18 | 3.42 | 0.00 | 0.00 |  |
| 19 | 3.33 | 0.00 | 0.00 | GO:0016491(oxidoreductase activity),GO:0050660(flavin adenine dinucleotide binding),GO:0016491(oxidoreductase activity),GO:0050660(flavin adenine dinucleotide binding) |
| 20 | 3.28 | 0.00 | 0.05 |  |
| 21 | 3.24 | 0.00 | 0.00 |  |
| 22 | 3.08 | 0.00 | 0.00 | GO:0004866(endopeptidase inhibitor activity) |
| 23 | 3.07 | 0.00 | 0.02 |  |
| 24 | 3.06 | 0.00 | 0.00 |  |
| 25 | 3.03 | 0.00 | 0.00 | GO:0004866(endopeptidase inhibitor activity) |
| 26 | 2.92 | 0.00 | 0.00 |  |
| 27 | 2.89 | 0.00 | 0.05 | GO:0016567(protein ubiquitination) |
| 28 | 2.89 | 0.00 | 0.00 | GO:0016787(hydrolase activity) |
| 29 | 2.88 | 0.00 | 0.00 | GO:0016491(oxidoreductase activity),GO:0050660(flavin adenine dinucleotide binding) |
| 30 | 2.76 | 0.00 | 0.00 | GO:0004866(endopeptidase inhibitor activity) |
| 31 | 2.74 | 0.00 | 0.00 |  |
| 32 | 2.70 | 0.00 | 0.00 |  |
| 33 | 2.67 | 0.00 | 0.00 |  |
| 34 | 2.64 | 0.00 | 0.00 |  |
| 35 | 2.58 | 0.00 | 0.00 |  |
| 36 | 2.56 | 0.00 | 0.00 |  |
| 37 | 2.51 | 0.00 | 0.00 | GO:0005515(protein binding) |
| 38 | 2.48 | 0.00 | 0.01 |  |
| 39 | 2.47 | 0.00 | 0.00 | GO:0016491(oxidoreductase activity),GO:0050660(flavin adenine dinucleotide binding) |
| 40 | 2.44 | 0.00 | 0.00 |  |
| 41 | 2.39 | 0.00 | 0.00 | GO:0006334(nucleosome assembly),GO:0000786(nucleosome) |
| 42 | 2.38 | 0.00 | 0.00 |  |
| 43 | 2.37 | 0.00 | 0.01 |  |
| 44 | 2.37 | 0.00 | 0.01 | GO:0009055(electron carrier activity),GO:0016705(oxidoreductase activity, acting on paired donors, with incorporation or reduction of molecular oxygen),GO:0020037(heme binding) |
| 45 | 2.36 | 0.00 | 0.03 |  |
| 46 | 2.34 | 0.00 | 0.00 |  |
| 47 | 2.32 | 0.00 | 0.04 | GO:0016491(oxidoreductase activity),GO:0050660(flavin adenine dinucleotide binding) |
| 48 | 2.31 | 0.00 | 0.01 |  |
| 49 | 2.25 | 0.00 | 0.02 |  |
| 50 | 2.23 | 0.00 | 0.01 |  |
| 51 | 2.22 | 0.00 | 0.00 | GO:0055114(oxidation-reduction process) |
| 52 | 2.21 | 0.00 | 0.03 |  |
| 53 | 2.21 | 0.00 | 0.00 |  |
| 54 | 2.19 | 0.00 | 0.02 | GO:0016829(lyase activity),GO:0008152(metabolic process) |
| 55 | 2.17 | 0.00 | 0.02 |  |
| 56 | 2.17 | 0.00 | 0.00 |  |
| 57 | 2.12 | 0.00 | 0.00 | GO:0006032(chitin catabolic process),GO:0016998(cell wall macromolecule catabolic process) |
| 58 | 2.11 | 0.00 | 0.00 |  |
| 59 | 2.11 | 0.00 | 0.05 |  |
| 60 | 2.11 | 0.00 | 0.00 |  |
| 61 | 2.11 | 0.00 | 0.00 |  |
| 62 | 2.08 | 0.00 | 0.00 |  |
| 63 | 2.06 | 0.00 | 0.00 |  |
| 64 | 2.05 | 0.00 | 0.00 | GO:0016829(lyase activity),GO:0008152(metabolic process) |
| 65 | 2.05 | 0.00 | 0.00 |  |
| 66 | 2.03 | 0.00 | 0.00 |  |
| 67 | 1.98 | 0.00 | 0.03 |  |
| 68 | 1.96 | 0.00 | 0.00 |  |
| 69 | 1.96 | 0.00 | 0.01 |  |
| 70 | 1.94 | 0.00 | 0.00 | GO:0000160(two-component signal transduction system (phosphorelay)),GO:0006355(regulation of transcription, DNA-dependent),GO:0000160(two-component signal transduction system (phosphorelay)),GO:0006355(regulation of transcription, DNA-dependent),GO:0000160(two-component signal transduction system (phosphorelay)),GO:0006355(regulation of transcription, DNA-dependent),GO:0000160(two-component signal transduction system (phosphorelay)),GO:0005515(protein binding),GO:0005515(protein binding),GO:0000160(two-component signal transduction system (phosphorelay)),GO:0000160(two-component signal transduction system (phosphorelay)),GO:0006355(regulation of transcription, DNA-dependent),GO:0000160(two-component signal transduction system (phosphorelay)),GO:0006355(regulation of transcription, DNA-dependent) |
| 71 | 1.93 | 0.00 | 0.00 | GO:0000160(two-component signal transduction system (phosphorelay)),GO:0006355(regulation of transcription, DNA-dependent),GO:0000160(two-component signal transduction system (phosphorelay)),GO:0006355(regulation of transcription, DNA-dependent),GO:0000160(two-component signal transduction system (phosphorelay)),GO:0006355(regulation of transcription, DNA-dependent),GO:0000160(two-component signal transduction system (phosphorelay)),GO:0005515(protein binding),GO:0005515(protein binding),GO:0000160(two-component signal transduction system (phosphorelay)),GO:0000160(two-component signal transduction system (phosphorelay)),GO:0006355(regulation of transcription, DNA-dependent),GO:0000160(two-component signal transduction system (phosphorelay)),GO:0006355(regulation of transcription, DNA-dependent) |
| 72 | 1.93 | 0.00 | 0.00 | GO:0000160(two-component signal transduction system (phosphorelay)),GO:0006355(regulation of transcription, DNA-dependent),GO:0000160(two-component signal transduction system (phosphorelay)),GO:0006355(regulation of transcription, DNA-dependent),GO:0000160(two-component signal transduction system (phosphorelay)),GO:0006355(regulation of transcription, DNA-dependent),GO:0000160(two-component signal transduction system (phosphorelay)),GO:0005515(protein binding),GO:0005515(protein binding),GO:0000160(two-component signal transduction system (phosphorelay)),GO:0000160(two-component signal transduction system (phosphorelay)),GO:0006355(regulation of transcription, DNA-dependent),GO:0000160(two-component signal transduction system (phosphorelay)),GO:0006355(regulation of transcription, DNA-dependent) |
| 73 | 1.92 | 0.00 | 0.01 |  |
| 74 | 1.92 | 0.00 | 0.00 | GO:0000160(two-component signal transduction system (phosphorelay)),GO:0006355(regulation of transcription, DNA-dependent),GO:0000160(two-component signal transduction system (phosphorelay)),GO:0006355(regulation of transcription, DNA-dependent),GO:0000160(two-component signal transduction system (phosphorelay)),GO:0006355(regulation of transcription, DNA-dependent),GO:0000160(two-component signal transduction system (phosphorelay)),GO:0005515(protein binding),GO:0005515(protein binding),GO:0000160(two-component signal transduction system (phosphorelay)),GO:0000160(two-component signal transduction system (phosphorelay)),GO:0006355(regulation of transcription, DNA-dependent),GO:0000160(two-component signal transduction system (phosphorelay)),GO:0006355(regulation of transcription, DNA-dependent) |
| 75 | 1.92 | 0.00 | 0.00 | GO:0000160(two-component signal transduction system (phosphorelay)),GO:0006355(regulation of transcription, DNA-dependent),GO:0000160(two-component signal transduction system (phosphorelay)),GO:0006355(regulation of transcription, DNA-dependent),GO:0000160(two-component signal transduction system (phosphorelay)),GO:0006355(regulation of transcription, DNA-dependent),GO:0000160(two-component signal transduction system (phosphorelay)),GO:0005515(protein binding),GO:0005515(protein binding),GO:0000160(two-component signal transduction system (phosphorelay)),GO:0000160(two-component signal transduction system (phosphorelay)),GO:0006355(regulation of transcription, DNA-dependent),GO:0000160(two-component signal transduction system (phosphorelay)),GO:0006355(regulation of transcription, DNA-dependent) |
| 76 | 1.91 | 0.00 | 0.00 | GO:0000160(two-component signal transduction system (phosphorelay)),GO:0006355(regulation of transcription, DNA-dependent),GO:0000160(two-component signal transduction system (phosphorelay)),GO:0006355(regulation of transcription, DNA-dependent),GO:0000160(two-component signal transduction system (phosphorelay)),GO:0006355(regulation of transcription, DNA-dependent),GO:0000160(two-component signal transduction system (phosphorelay)),GO:0005515(protein binding),GO:0005515(protein binding),GO:0000160(two-component signal transduction system (phosphorelay)),GO:0000160(two-component signal transduction system (phosphorelay)),GO:0006355(regulation of transcription, DNA-dependent),GO:0000160(two-component signal transduction system (phosphorelay)),GO:0006355(regulation of transcription, DNA-dependent) |
| 77 | 1.91 | 0.00 | 0.00 | GO:0000160(two-component signal transduction system (phosphorelay)),GO:0006355(regulation of transcription, DNA-dependent),GO:0000160(two-component signal transduction system (phosphorelay)),GO:0006355(regulation of transcription, DNA-dependent),GO:0000160(two-component signal transduction system (phosphorelay)),GO:0006355(regulation of transcription, DNA-dependent),GO:0000160(two-component signal transduction system (phosphorelay)),GO:0005515(protein binding),GO:0005515(protein binding),GO:0000160(two-component signal transduction system (phosphorelay)),GO:0000160(two-component signal transduction system (phosphorelay)),GO:0006355(regulation of transcription, DNA-dependent),GO:0000160(two-component signal transduction system (phosphorelay)),GO:0006355(regulation of transcription, DNA-dependent) |
| 78 | 1.90 | 0.00 | 0.00 | GO:0000160(two-component signal transduction system (phosphorelay)),GO:0006355(regulation of transcription, DNA-dependent),GO:0000160(two-component signal transduction system (phosphorelay)),GO:0006355(regulation of transcription, DNA-dependent),GO:0000160(two-component signal transduction system (phosphorelay)),GO:0006355(regulation of transcription, DNA-dependent),GO:0000160(two-component signal transduction system (phosphorelay)),GO:0005515(protein binding),GO:0005515(protein binding),GO:0000160(two-component signal transduction system (phosphorelay)),GO:0000160(two-component signal transduction system (phosphorelay)),GO:0006355(regulation of transcription, DNA-dependent),GO:0000160(two-component signal transduction system (phosphorelay)),GO:0006355(regulation of transcription, DNA-dependent) |
| 79 | 1.89 | 0.00 | 0.00 | GO:0000160(two-component signal transduction system (phosphorelay)),GO:0006355(regulation of transcription, DNA-dependent),GO:0000160(two-component signal transduction system (phosphorelay)),GO:0006355(regulation of transcription, DNA-dependent),GO:0000160(two-component signal transduction system (phosphorelay)),GO:0006355(regulation of transcription, DNA-dependent),GO:0000160(two-component signal transduction system (phosphorelay)),GO:0005515(protein binding),GO:0005515(protein binding),GO:0000160(two-component signal transduction system (phosphorelay)),GO:0000160(two-component signal transduction system (phosphorelay)),GO:0006355(regulation of transcription, DNA-dependent),GO:0000160(two-component signal transduction system (phosphorelay)),GO:0006355(regulation of transcription, DNA-dependent) |
| 80 | 1.89 | 0.00 | 0.01 | GO:0030170(pyridoxal phosphate binding),GO:0009058(biosynthetic process),GO:0030170(pyridoxal phosphate binding),GO:0009058(biosynthetic process) |
| 81 | 1.87 | 0.00 | 0.00 |  |
| 82 | 1.87 | 0.00 | 0.01 |  |
| 83 | 1.86 | 0.00 | 0.00 | GO:0016757(transferase activity, transferring glycosyl groups),GO:0016757(transferase activity, transferring glycosyl groups),GO:0016757(transferase activity, transferring glycosyl groups) |
| 84 | 1.86 | 0.00 | 0.01 | GO:0005524(ATP binding) |
| 85 | 1.86 | 0.00 | 0.05 | GO:0044237(cellular metabolic process) |
| 86 | 1.85 | 0.00 | 0.00 | GO:0016757(transferase activity, transferring glycosyl groups),GO:0016757(transferase activity, transferring glycosyl groups),GO:0016757(transferase activity, transferring glycosyl groups) |
| 87 | 1.85 | 0.00 | 0.00 | GO:0016757(transferase activity, transferring glycosyl groups),GO:0016757(transferase activity, transferring glycosyl groups),GO:0016757(transferase activity, transferring glycosyl groups) |
| 88 | 1.84 | 0.00 | 0.04 |  |
| 89 | 1.82 | 0.00 | 0.00 |  |
| 90 | 1.82 | 0.00 | 0.01 |  |
| 91 | 1.82 | 0.00 | 0.01 | GO:0030170(pyridoxal phosphate binding),GO:0009058(biosynthetic process),GO:0030170(pyridoxal phosphate binding),GO:0009058(biosynthetic process) |
| 92 | 1.82 | 0.00 | 0.00 |  |
| 93 | 1.81 | 0.00 | 0.02 | GO:0005524(ATP binding),GO:0006468(protein phosphorylation) |
| 94 | 1.81 | 0.00 | 0.02 | GO:0016491(oxidoreductase activity),GO:0050660(flavin adenine dinucleotide binding) |
| 95 | 1.81 | 0.00 | 0.00 |  |
| 96 | 1.80 | 0.00 | 0.04 |  |
| 97 | 1.80 | 0.00 | 0.00 |  |
| 98 | 1.80 | 0.00 | 0.05 |  |
| 99 | 1.79 | 0.00 | 0.00 |  |
| 100 | 1.78 | 0.00 | 0.01 |  |
| 101 | 1.77 | 0.00 | 0.02 | GO:0003824(catalytic activity),GO:0003824(catalytic activity) |
| 102 | 1.77 | 0.00 | 0.02 | GO:0003824(catalytic activity),GO:0003824(catalytic activity) |
| 103 | 1.77 | 0.00 | 0.02 | GO:0003824(catalytic activity) |
| 104 | 1.76 | 0.00 | 0.01 |  |
| 105 | 1.76 | 0.00 | 0.00 |  |
| 106 | 1.76 | 0.00 | 0.00 |  |
| 107 | 1.76 | 0.00 | 0.01 |  |
| 108 | 1.76 | 0.00 | 0.02 |  |
| 109 | 1.75 | 0.00 | 0.05 |  |
| 110 | 1.75 | 0.00 | 0.02 |  |
| 111 | 1.74 | 0.00 | 0.00 |  |
| 112 | 1.71 | 0.00 | 0.01 | GO:0005524(ATP binding),GO:0006468(protein phosphorylation),GO:0048544(recognition of pollen) |
| 113 | 1.71 | 0.00 | 0.03 |  |
| 114 | 1.69 | 0.00 | 0.05 | GO:0005515(protein binding),GO:0005515(protein binding) |
| 115 | 1.67 | 0.00 | 0.01 |  |
| 116 | 1.67 | 0.00 | 0.01 |  |
| 117 | 1.67 | 0.00 | 0.04 | GO:0055085(transmembrane transport),GO:0055085(transmembrane transport) |
| 118 | 1.66 | 0.00 | 0.04 |  |
| 119 | 1.65 | 0.00 | 0.01 |  |
| 120 | 1.65 | 0.00 | 0.01 |  |
| 121 | 1.64 | 0.00 | 0.05 | GO:0016021(integral to membrane),GO:0016020(membrane),GO:0016021(integral to membrane),GO:0016020(membrane),GO:0016021(integral to membrane),GO:0016020(membrane),GO:0016021(integral to membrane),GO:0016020(membrane),GO:0016021(integral to membrane),GO:0016020(membrane),GO:0016021(integral to membrane),GO:0016020(membrane),GO:0016021(integral to membrane),GO:0016020(membrane) |
| 122 | 1.64 | 0.00 | 0.05 | GO:0055114(oxidation-reduction process) |
| 123 | 1.63 | 0.00 | 0.03 |  |
| 124 | 1.61 | 0.00 | 0.01 | GO:0016706(oxidoreductase activity, acting on paired donors, with incorporation or reduction of molecular oxygen, 2-oxoglutarate as one donor, and incorporation of one atom each of oxygen into both donors),GO:0016706(oxidoreductase activity, acting on paired donors, with incorporation or reduction of molecular oxygen, 2-oxoglutarate as one donor, and incorporation of one atom each of oxygen into both donors) |
| 125 | 1.61 | 0.00 | 0.01 |  |
| 126 | 1.60 | 0.00 | 0.01 | GO:0003677(DNA binding),GO:0003677(DNA binding) |
| 127 | 1.59 | 0.00 | 0.02 |  |
| 128 | 1.59 | 0.00 | 0.01 | GO:0016706(oxidoreductase activity, acting on paired donors, with incorporation or reduction of molecular oxygen, 2-oxoglutarate as one donor, and incorporation of one atom each of oxygen into both donors),GO:0016706(oxidoreductase activity, acting on paired donors, with incorporation or reduction of molecular oxygen, 2-oxoglutarate as one donor, and incorporation of one atom each of oxygen into both donors) |
| 129 | 1.58 | 0.00 | 0.02 |  |
| 130 | 1.58 | 0.00 | 0.02 |  |
| 131 | 1.58 | 0.00 | 0.02 | GO:0006629(lipid metabolic process),GO:0055114(oxidation-reduction process) |
| 132 | 1.55 | 0.00 | 0.02 | GO:0003677(DNA binding),GO:0003677(DNA binding) |
| 133 | 1.55 | 0.00 | 0.02 |  |
| 134 | 1.54 | 0.00 | 0.04 | GO:0016491(oxidoreductase activity),GO:0050660(flavin adenine dinucleotide binding) |
| 135 | 1.53 | 0.00 | 0.03 | GO:0003677(DNA binding),GO:0003677(DNA binding) |
| 136 | 1.53 | 0.00 | 0.03 | GO:0016706(oxidoreductase activity, acting on paired donors, with incorporation or reduction of molecular oxygen, 2-oxoglutarate as one donor, and incorporation of one atom each of oxygen into both donors) |
| 137 | 1.52 | 0.00 | 0.03 |  |
| 138 | 1.52 | 0.00 | 0.04 | GO:0006352(transcription initiation, DNA-dependent),GO:0005634(nucleus),GO:0006334(nucleosome assembly) |
| 139 | 1.51 | 0.00 | 0.04 | GO:0016717(oxidoreductase activity, acting on paired donors, with oxidation of a pair of donors resulting in the reduction of molecular oxygen to two molecules of water),GO:0055114(oxidation-reduction process),GO:0016717(oxidoreductase activity, acting on paired donors, with oxidation of a pair of donors resulting in the reduction of molecular oxygen to two molecules of water),GO:0055114(oxidation-reduction process),GO:0016717(oxidoreductase activity, acting on paired donors, with oxidation of a pair of donors resulting in the reduction of molecular oxygen to two molecules of water),GO:0055114(oxidation-reduction process) |
| 140 | 1.51 | 0.00 | 0.04 |  |
| 141 | 1.50 | 0.00 | 0.04 |  |
| 142 | 1.50 | 0.00 | 0.04 |  |
| 143 | 1.50 | 0.00 | 0.05 |  |
| 144 | 1.50 | 0.00 | 0.05 |  |
| 145 | 1.50 | 0.00 | 0.04 | GO:0006021(inositol biosynthetic process),GO:0006021(inositol biosynthetic process),GO:0006021(inositol biosynthetic process),GO:0006021(inositol biosynthetic process),GO:0006021(inositol biosynthetic process),GO:0006021(inositol biosynthetic process),GO:0006021(inositol biosynthetic process),GO:0006021(inositol biosynthetic process),GO:0006021(inositol biosynthetic process),GO:0006021(inositol biosynthetic process),GO:0006021(inositol biosynthetic process),GO:0006021(inositol biosynthetic process) |
| 146 | 1.50 | 0.00 | 0.04 | GO:0006021(inositol biosynthetic process),GO:0006021(inositol biosynthetic process),GO:0006021(inositol biosynthetic process),GO:0006021(inositol biosynthetic process),GO:0006021(inositol biosynthetic process),GO:0006021(inositol biosynthetic process),GO:0006021(inositol biosynthetic process),GO:0006021(inositol biosynthetic process),GO:0006021(inositol biosynthetic process),GO:0006021(inositol biosynthetic process),GO:0006021(inositol biosynthetic process),GO:0006021(inositol biosynthetic process) |
| 147 | 1.50 | 0.00 | 0.04 | GO:0016706(oxidoreductase activity, acting on paired donors, with incorporation or reduction of molecular oxygen, 2-oxoglutarate as one donor, and incorporation of one atom each of oxygen into both donors) |
| 148 | 1.50 | 0.00 | 0.04 |  |
| 149 | 1.49 | 0.00 | 0.04 | GO:0006021(inositol biosynthetic process),GO:0006021(inositol biosynthetic process),GO:0006021(inositol biosynthetic process),GO:0006021(inositol biosynthetic process),GO:0006021(inositol biosynthetic process),GO:0006021(inositol biosynthetic process),GO:0006021(inositol biosynthetic process),GO:0006021(inositol biosynthetic process),GO:0006021(inositol biosynthetic process),GO:0006021(inositol biosynthetic process),GO:0006021(inositol biosynthetic process),GO:0006021(inositol biosynthetic process) |
| 150 | 1.49 | 0.00 | 0.03 |  |
| 151 | 1.49 | 0.00 | 0.04 | GO:0006021(inositol biosynthetic process),GO:0006021(inositol biosynthetic process),GO:0006021(inositol biosynthetic process),GO:0006021(inositol biosynthetic process),GO:0006021(inositol biosynthetic process),GO:0006021(inositol biosynthetic process),GO:0006021(inositol biosynthetic process),GO:0006021(inositol biosynthetic process),GO:0006021(inositol biosynthetic process),GO:0006021(inositol biosynthetic process),GO:0006021(inositol biosynthetic process),GO:0006021(inositol biosynthetic process) |
| 152 | 1.49 | 0.00 | 0.04 | GO:0030170(pyridoxal phosphate binding),GO:0009058(biosynthetic process),GO:0008152(metabolic process) |
| 153 | 1.49 | 0.00 | 0.04 | GO:0006021(inositol biosynthetic process),GO:0006021(inositol biosynthetic process),GO:0006021(inositol biosynthetic process),GO:0006021(inositol biosynthetic process),GO:0006021(inositol biosynthetic process),GO:0006021(inositol biosynthetic process),GO:0006021(inositol biosynthetic process),GO:0006021(inositol biosynthetic process),GO:0006021(inositol biosynthetic process),GO:0006021(inositol biosynthetic process),GO:0006021(inositol biosynthetic process),GO:0006021(inositol biosynthetic process) |
| 154 | 1.48 | 0.00 | 0.05 |  |
| 155 | 1.48 | 0.00 | 0.04 | GO:0006021(inositol biosynthetic process),GO:0006021(inositol biosynthetic process),GO:0006021(inositol biosynthetic process),GO:0006021(inositol biosynthetic process),GO:0006021(inositol biosynthetic process),GO:0006021(inositol biosynthetic process),GO:0006021(inositol biosynthetic process),GO:0006021(inositol biosynthetic process),GO:0006021(inositol biosynthetic process),GO:0006021(inositol biosynthetic process),GO:0006021(inositol biosynthetic process),GO:0006021(inositol biosynthetic process) |
| 156 | 1.48 | 0.00 | 0.04 | GO:0006021(inositol biosynthetic process),GO:0006021(inositol biosynthetic process),GO:0006021(inositol biosynthetic process),GO:0006021(inositol biosynthetic process),GO:0006021(inositol biosynthetic process),GO:0006021(inositol biosynthetic process),GO:0006021(inositol biosynthetic process),GO:0006021(inositol biosynthetic process),GO:0006021(inositol biosynthetic process),GO:0006021(inositol biosynthetic process),GO:0006021(inositol biosynthetic process),GO:0006021(inositol biosynthetic process) |
| 157 | 1.48 | 0.00 | 0.04 |  |
| 158 | 1.48 | 0.00 | 0.04 | GO:0006021(inositol biosynthetic process),GO:0006021(inositol biosynthetic process),GO:0006021(inositol biosynthetic process),GO:0006021(inositol biosynthetic process),GO:0006021(inositol biosynthetic process),GO:0006021(inositol biosynthetic process),GO:0006021(inositol biosynthetic process),GO:0006021(inositol biosynthetic process),GO:0006021(inositol biosynthetic process),GO:0006021(inositol biosynthetic process),GO:0006021(inositol biosynthetic process),GO:0006021(inositol biosynthetic process) |
| 159 | 1.48 | 0.00 | 0.04 | GO:0006021(inositol biosynthetic process),GO:0006021(inositol biosynthetic process),GO:0006021(inositol biosynthetic process),GO:0006021(inositol biosynthetic process),GO:0006021(inositol biosynthetic process),GO:0006021(inositol biosynthetic process),GO:0006021(inositol biosynthetic process),GO:0006021(inositol biosynthetic process),GO:0006021(inositol biosynthetic process),GO:0006021(inositol biosynthetic process),GO:0006021(inositol biosynthetic process),GO:0006021(inositol biosynthetic process) |
| 160 | 1.47 | 0.00 | 0.05 |  |
| 161 | 1.47 | 0.00 | 0.04 | GO:0006021(inositol biosynthetic process),GO:0006021(inositol biosynthetic process),GO:0006021(inositol biosynthetic process),GO:0006021(inositol biosynthetic process),GO:0006021(inositol biosynthetic process),GO:0006021(inositol biosynthetic process),GO:0006021(inositol biosynthetic process),GO:0006021(inositol biosynthetic process),GO:0006021(inositol biosynthetic process),GO:0006021(inositol biosynthetic process),GO:0006021(inositol biosynthetic process),GO:0006021(inositol biosynthetic process) |
| 162 | 1.47 | 0.00 | 0.04 | GO:0016829(lyase activity),GO:0008152(metabolic process),GO:0016829(lyase activity),GO:0008152(metabolic process) |
| 163 | 1.47 | 0.00 | 0.04 |  |
| 164 | 1.47 | 0.00 | 0.04 | GO:0006021(inositol biosynthetic process),GO:0006021(inositol biosynthetic process),GO:0006021(inositol biosynthetic process),GO:0006021(inositol biosynthetic process),GO:0006021(inositol biosynthetic process),GO:0006021(inositol biosynthetic process),GO:0006021(inositol biosynthetic process),GO:0006021(inositol biosynthetic process),GO:0006021(inositol biosynthetic process),GO:0006021(inositol biosynthetic process),GO:0006021(inositol biosynthetic process),GO:0006021(inositol biosynthetic process) |
| 165 | 1.47 | 0.00 | 0.04 |  |
| 166 | 1.47 | 0.00 | 0.04 |  |
| 167 | 1.47 | 0.00 | 0.05 | GO:0006021(inositol biosynthetic process),GO:0006021(inositol biosynthetic process),GO:0006021(inositol biosynthetic process),GO:0006021(inositol biosynthetic process),GO:0006021(inositol biosynthetic process),GO:0006021(inositol biosynthetic process),GO:0006021(inositol biosynthetic process),GO:0006021(inositol biosynthetic process),GO:0006021(inositol biosynthetic process),GO:0006021(inositol biosynthetic process),GO:0006021(inositol biosynthetic process),GO:0006021(inositol biosynthetic process) |
| 168 | 1.43 | 0.00 | 0.05 |  |
| 169 | 1.43 | 0.00 | 0.05 | GO:0016829(lyase activity),GO:0008152(metabolic process),GO:0016829(lyase activity),GO:0008152(metabolic process) |
| 170 | 1.43 | 0.00 | 0.05 |  |
| 171 | 1.43 | 0.00 | 0.05 |  |
| 172 | -1.44 | 0.00 | 0.05 | GO:0043565(sequence-specific DNA binding),GO:0006355(regulation of transcription, DNA-dependent),GO:0003677(DNA binding),GO:0005634(nucleus),GO:0006351(transcription, DNA-dependent) |
| 173 | -1.45 | 0.00 | 0.05 | GO:0005509(calcium ion binding),GO:0005509(calcium ion binding) |
| 174 | -1.47 | 0.00 | 0.05 |  |
| 175 | -1.50 | 0.00 | 0.05 | GO:0031072(heat shock protein binding),GO:0031072(heat shock protein binding),GO:0031072(heat shock protein binding),GO:0031072(heat shock protein binding),GO:0031072(heat shock protein binding) |
| 176 | -1.51 | 0.00 | 0.03 | GO:0005524(ATP binding),GO:0006468(protein phosphorylation),GO:0016773(phosphotransferase activity, alcohol group as acceptor),GO:0009103(lipopolysaccharide biosynthetic process) |
| 177 | -1.55 | 0.00 | 0.05 | GO:0009055(electron carrier activity),GO:0016705(oxidoreductase activity, acting on paired donors, with incorporation or reduction of molecular oxygen),GO:0020037(heme binding) |
| 178 | -1.55 | 0.00 | 0.02 | GO:0006629(lipid metabolic process),GO:0006629(lipid metabolic process),GO:0006629(lipid metabolic process) |
| 179 | -1.56 | 0.00 | 0.02 | GO:0003676(nucleic acid binding),GO:0003676(nucleic acid binding) |
| 180 | -1.57 | 0.00 | 0.02 | GO:0006915(apoptotic process) |
| 181 | -1.58 | 0.00 | 0.05 | GO:0008977(prephenate dehydrogenase activity),GO:0006571(tyrosine biosynthetic process),GO:0055114(oxidation-reduction process),GO:0008977(prephenate dehydrogenase activity),GO:0006571(tyrosine biosynthetic process),GO:0055114(oxidation-reduction process),GO:0008977(prephenate dehydrogenase activity),GO:0006571(tyrosine biosynthetic process),GO:0055114(oxidation-reduction process) |
| 182 | -1.62 | 0.00 | 0.04 | GO:0008977(prephenate dehydrogenase activity),GO:0006571(tyrosine biosynthetic process),GO:0055114(oxidation-reduction process),GO:0008977(prephenate dehydrogenase activity),GO:0006571(tyrosine biosynthetic process),GO:0055114(oxidation-reduction process),GO:0008977(prephenate dehydrogenase activity),GO:0006571(tyrosine biosynthetic process),GO:0055114(oxidation-reduction process) |
| 183 | -1.63 | 0.00 | 0.05 |  |
| 184 | -1.63 | 0.00 | 0.05 | GO:0006629(lipid metabolic process) |
| 185 | -1.65 | 0.00 | 0.05 |  |
| 186 | -1.65 | 0.00 | 0.01 |  |
| 187 | -1.66 | 0.00 | 0.04 | GO:0016706(oxidoreductase activity, acting on paired donors, with incorporation or reduction of molecular oxygen, 2-oxoglutarate as one donor, and incorporation of one atom each of oxygen into both donors) |
| 188 | -1.67 | 0.00 | 0.01 |  |
| 189 | -1.67 | 0.00 | 0.03 |  |
| 190 | -1.67 | 0.00 | 0.01 | GO:0003677(DNA binding) |
| 191 | -1.67 | 0.00 | 0.01 | GO:0006950(response to stress) |
| 192 | -1.69 | 0.00 | 0.01 |  |
| 193 | -1.69 | 0.00 | 0.02 |  |
| 194 | -1.71 | 0.00 | 0.01 | GO:0051082(unfolded protein binding),GO:0006457(protein folding) |
| 195 | -1.72 | 0.00 | 0.03 |  |
| 196 | -1.72 | 0.00 | 0.00 | GO:0005515(protein binding) |
| 197 | -1.72 | 0.00 | 0.02 | GO:0043565(sequence-specific DNA binding),GO:0006355(regulation of transcription, DNA-dependent),GO:0043565(sequence-specific DNA binding),GO:0006355(regulation of transcription, DNA-dependent),GO:0043565(sequence-specific DNA binding),GO:0006355(regulation of transcription, DNA-dependent) |
| 198 | -1.72 | 0.00 | 0.01 | GO:0005509(calcium ion binding),GO:0005509(calcium ion binding) |
| 199 | -1.72 | 0.00 | 0.01 | GO:0006629(lipid metabolic process),GO:0006629(lipid metabolic process),GO:0006629(lipid metabolic process) |
| 200 | -1.72 | 0.00 | 0.01 |  |
| 201 | -1.73 | 0.00 | 0.00 | GO:0005515(protein binding) |
| 202 | -1.73 | 0.00 | 0.04 |  |
| 203 | -1.73 | 0.00 | 0.04 |  |
| 204 | -1.74 | 0.00 | 0.02 |  |
| 205 | -1.75 | 0.00 | 0.01 |  |
| 206 | -1.75 | 0.00 | 0.00 | GO:0016706(oxidoreductase activity, acting on paired donors, with incorporation or reduction of molecular oxygen, 2-oxoglutarate as one donor, and incorporation of one atom each of oxygen into both donors),GO:0016706(oxidoreductase activity, acting on paired donors, with incorporation or reduction of molecular oxygen, 2-oxoglutarate as one donor, and incorporation of one atom each of oxygen into both donors) |
| 207 | -1.76 | 0.00 | 0.01 | GO:0043565(sequence-specific DNA binding),GO:0006355(regulation of transcription, DNA-dependent),GO:0043565(sequence-specific DNA binding),GO:0006355(regulation of transcription, DNA-dependent),GO:0043565(sequence-specific DNA binding),GO:0006355(regulation of transcription, DNA-dependent) |
| 208 | -1.78 | 0.00 | 0.00 | GO:0016706(oxidoreductase activity, acting on paired donors, with incorporation or reduction of molecular oxygen, 2-oxoglutarate as one donor, and incorporation of one atom each of oxygen into both donors),GO:0016706(oxidoreductase activity, acting on paired donors, with incorporation or reduction of molecular oxygen, 2-oxoglutarate as one donor, and incorporation of one atom each of oxygen into both donors) |
| 209 | -1.80 | 0.00 | 0.01 |  |
| 210 | -1.81 | 0.00 | 0.00 | GO:0043565(sequence-specific DNA binding),GO:0006355(regulation of transcription, DNA-dependent),GO:0003677(DNA binding),GO:0005634(nucleus),GO:0006351(transcription, DNA-dependent),GO:0043565(sequence-specific DNA binding),GO:0006355(regulation of transcription, DNA-dependent),GO:0003677(DNA binding),GO:0005634(nucleus),GO:0006351(transcription, DNA-dependent),GO:0043565(sequence-specific DNA binding),GO:0006355(regulation of transcription, DNA-dependent),GO:0003677(DNA binding),GO:0005634(nucleus),GO:0006351(transcription, DNA-dependent) |
| 211 | -1.81 | 0.00 | 0.00 | GO:0009055(electron carrier activity),GO:0016705(oxidoreductase activity, acting on paired donors, with incorporation or reduction of molecular oxygen),GO:0020037(heme binding) |
| 212 | -1.82 | 0.00 | 0.00 |  |
| 213 | -1.83 | 0.00 | 0.00 |  |
| 214 | -1.83 | 0.00 | 0.00 | GO:0043565(sequence-specific DNA binding),GO:0006355(regulation of transcription, DNA-dependent),GO:0003677(DNA binding),GO:0005634(nucleus),GO:0006351(transcription, DNA-dependent),GO:0043565(sequence-specific DNA binding),GO:0006355(regulation of transcription, DNA-dependent),GO:0003677(DNA binding),GO:0005634(nucleus),GO:0006351(transcription, DNA-dependent),GO:0043565(sequence-specific DNA binding),GO:0006355(regulation of transcription, DNA-dependent),GO:0003677(DNA binding),GO:0005634(nucleus),GO:0006351(transcription, DNA-dependent) |
| 215 | -1.84 | 0.00 | 0.00 | GO:0043565(sequence-specific DNA binding),GO:0006355(regulation of transcription, DNA-dependent),GO:0003677(DNA binding),GO:0005634(nucleus),GO:0006351(transcription, DNA-dependent),GO:0043565(sequence-specific DNA binding),GO:0006355(regulation of transcription, DNA-dependent),GO:0003677(DNA binding),GO:0005634(nucleus),GO:0006351(transcription, DNA-dependent),GO:0043565(sequence-specific DNA binding),GO:0006355(regulation of transcription, DNA-dependent),GO:0003677(DNA binding),GO:0005634(nucleus),GO:0006351(transcription, DNA-dependent) |
| 216 | -1.85 | 0.00 | 0.00 |  |
| 217 | -1.86 | 0.00 | 0.00 |  |
| 218 | -1.87 | 0.00 | 0.00 | GO:0043565(sequence-specific DNA binding),GO:0006355(regulation of transcription, DNA-dependent),GO:0043565(sequence-specific DNA binding),GO:0006355(regulation of transcription, DNA-dependent),GO:0043565(sequence-specific DNA binding),GO:0006355(regulation of transcription, DNA-dependent) |
| 219 | -1.87 | 0.00 | 0.00 |  |
| 220 | -1.87 | 0.00 | 0.00 |  |
| 221 | -1.87 | 0.00 | 0.00 |  |
| 222 | -1.87 | 0.00 | 0.00 | GO:0006629(lipid metabolic process),GO:0006629(lipid metabolic process),GO:0006629(lipid metabolic process) |
| 223 | -1.88 | 0.00 | 0.05 | GO:0016491(oxidoreductase activity),GO:0016491(oxidoreductase activity) |
| 224 | -1.92 | 0.00 | 0.00 |  |
| 225 | -1.94 | 0.00 | 0.05 | GO:0003676(nucleic acid binding),GO:0003676(nucleic acid binding),GO:0003676(nucleic acid binding),GO:0003676(nucleic acid binding),GO:0003676(nucleic acid binding) |
| 226 | -1.94 | 0.00 | 0.04 | GO:0003676(nucleic acid binding),GO:0003676(nucleic acid binding),GO:0003676(nucleic acid binding),GO:0003676(nucleic acid binding),GO:0003676(nucleic acid binding) |
| 227 | -1.97 | 0.00 | 0.00 |  |
| 228 | -2.00 | 0.00 | 0.00 |  |
| 229 | -2.02 | 0.00 | 0.02 | GO:0015035(protein disulfide oxidoreductase activity),GO:0009055(electron carrier activity),GO:0015035(protein disulfide oxidoreductase activity),GO:0009055(electron carrier activity),GO:0015035(protein disulfide oxidoreductase activity),GO:0009055(electron carrier activity) |
| 230 | -2.03 | 0.00 | 0.00 |  |
| 231 | -2.06 | 0.00 | 0.00 |  |
| 232 | -2.06 | 0.00 | 0.00 |  |
| 233 | -2.07 | 0.00 | 0.00 |  |
| 234 | -2.09 | 0.00 | 0.00 |  |
| 235 | -2.11 | 0.00 | 0.00 |  |
| 236 | -2.11 | 0.00 | 0.00 |  |
| 237 | -2.12 | 0.00 | 0.00 |  |
| 238 | -2.15 | 0.00 | 0.00 |  |
| 239 | -2.16 | 0.00 | 0.00 | GO:0009055(electron carrier activity),GO:0016705(oxidoreductase activity, acting on paired donors, with incorporation or reduction of molecular oxygen),GO:0020037(heme binding) |
| 240 | -2.16 | 0.00 | 0.02 | GO:0003676(nucleic acid binding),GO:0003676(nucleic acid binding),GO:0003676(nucleic acid binding),GO:0003676(nucleic acid binding),GO:0003676(nucleic acid binding) |
| 241 | -2.17 | 0.00 | 0.01 |  |
| 242 | -2.17 | 0.00 | 0.00 |  |
| 243 | -2.20 | 0.00 | 0.01 |  |
| 244 | -2.36 | 0.00 | 0.00 | GO:0051082(unfolded protein binding),GO:0006457(protein folding),GO:0051082(unfolded protein binding),GO:0006457(protein folding) |
| 245 | -2.38 | 0.00 | 0.00 | GO:0051082(unfolded protein binding),GO:0006457(protein folding),GO:0051082(unfolded protein binding),GO:0006457(protein folding) |
| 246 | -2.46 | 0.00 | 0.00 | GO:0004563(beta-N-acetylhexosaminidase activity) |
| 247 | -2.53 | 0.00 | 0.00 |  |
| 248 | -2.56 | 0.00 | 0.00 |  |
| 249 | -2.57 | 0.00 | 0.00 |  |
| 250 | -2.59 | 0.00 | 0.00 | GO:0009055(electron carrier activity),GO:0016705(oxidoreductase activity, acting on paired donors, with incorporation or reduction of molecular oxygen),GO:0020037(heme binding) |
| 251 | -2.60 | 0.00 | 0.00 | GO:0005515(protein binding),GO:0005515(protein binding) |
| 252 | -2.62 | 0.00 | 0.00 | GO:0004563(beta-N-acetylhexosaminidase activity) |
| 253 | -2.72 | 0.00 | 0.00 | GO:0005515(protein binding),GO:0005515(protein binding) |
| 254 | -2.74 | 0.00 | 0.00 | GO:0006457(protein folding) |
| 255 | -2.75 | 0.00 | 0.00 | GO:0006457(protein folding) |
| 256 | -2.78 | 0.00 | 0.00 | GO:0006457(protein folding) |
| 257 | -2.78 | 0.00 | 0.00 | GO:0006457(protein folding) |
| 258 | -2.81 | 0.00 | 0.01 |  |
| 259 | -2.82 | 0.00 | 0.00 |  |
| 260 | -2.85 | 0.00 | 0.05 | GO:0016706(oxidoreductase activity, acting on paired donors, with incorporation or reduction of molecular oxygen, 2-oxoglutarate as one donor, and incorporation of one atom each of oxygen into both donors) |
| 261 | -2.92 | 0.00 | 0.04 | GO:0005515(protein binding),GO:0005515(protein binding),GO:0005515(protein binding),GO:0005515(protein binding) |
| 262 | -3.01 | 0.00 | 0.00 |  |
| 263 | -3.10 | 0.00 | 0.01 | GO:0005515(protein binding),GO:0005515(protein binding),GO:0005515(protein binding),GO:0005515(protein binding) |
| 264 | -3.10 | 0.00 | 0.01 | GO:0005515(protein binding),GO:0005515(protein binding),GO:0005515(protein binding),GO:0005515(protein binding) |
| 265 | -3.10 | 0.00 | 0.01 | GO:0005515(protein binding),GO:0005515(protein binding),GO:0005515(protein binding),GO:0005515(protein binding) |
| 266 | -3.21 | 0.00 | 0.00 | GO:0016747(transferase activity, transferring acyl groups other than amino-acyl groups) |
| 267 | -3.41 | 0.00 | 0.00 | GO:0009055(electron carrier activity),GO:0016705(oxidoreductase activity, acting on paired donors, with incorporation or reduction of molecular oxygen),GO:0020037(heme binding) |
| 268 | -3.50 | 0.00 | 0.00 |  |
| 269 | -3.53 | 0.00 | 0.00 |  |
| 270 | -3.57 | 0.00 | 0.00 |  |
| 271 | -3.78 | 0.00 | 0.00 |  |
| 272 | -4.04 | 0.00 | 0.05 |  |
| 273 | -4.69 | 0.00 | 0.00 |  |
| 274 | -4.85 | 0.00 | 0.00 | GO:0009055(electron carrier activity),GO:0016705(oxidoreductase activity, acting on paired donors, with incorporation or reduction of molecular oxygen),GO:0020037(heme binding) |
| 275 | -5.17 | 0.00 | 0.00 | GO:0005515(protein binding) |
| 276 | -5.29 | 0.00 | 0.00 |  |
| 277 | -5.60 | 0.00 | 0.00 |  |
| 278 | -5.88 | 0.00 | 0.00 | GO:0030170(pyridoxal phosphate binding),GO:0030170(pyridoxal phosphate binding) |
| 279 | -6.04 | 0.00 | 0.00 | GO:0030170(pyridoxal phosphate binding),GO:0030170(pyridoxal phosphate binding) |
| 280 | -16.61 | 0.00 | 0.01 |  |
| 281 | -16.61 | 0.00 | 0.00 | GO:0009055(electron carrier activity),GO:0016705(oxidoreductase activity, acting on paired donors, with incorporation or reduction of molecular oxygen),GO:0020037(heme binding) |
| 282 | -16.61 | 0.00 | 0.01 |  |

Table S6 miRNA and target genes expression level at different time points post inoculation

| **miR** | **Log** | **gp** | **Target** | **Target gene annomination** |
| --- | --- | --- | --- | --- |
| **miR172g-3p** | -1.02 | gp1a | 0.66 | AP2/ERF superfamily transcription factor |
| **miR171i-p3** | -2.04 | gp2a | 0.22 | GRAS family transcription factor gene |
| **miR393_R+2** | 1.64 | gp2a | -0.23 | F-box/RNI-like superfamily gene |
| **miR828a** | -1.04 | gp1a | -0.21573 | MYB transcription factor gene |
| **miR172b-5p** | -2.63 | gp1a | -0.06 | WRKY70 gene |
| **miR482a** | -1.25 | gp2a | 0.5 | TIR-NBS-LRR gene |
| **miR5032-p3** | 1.63 | gp1b | 0.02 | GroES-like zinc-binding dehydrogenase family protein gene |
| **miR6485-p3** | 1.33 | gp1b | -0.61 | O-methyltransferases gene |
| **miR171e-p5** | 1.77 | gp1a | 0.54 | epsin N-terminal homology (ENTH) domain-containing protein gene |
| **miR6468** | 2.77 | gp1a | -0.79 | HXXXD-type acyl-transferase family gene |

Table S7 Poplar defense pathway miRNA and target genes

| **miRNA** | **Log_2_ miRNA** | **Target genes** | **Log_2_ target genes** |
| --- | --- | --- | --- |
|  |  | RLK |  |
| **PC-5p-359200_2** | -10.88 | Cysteine-rich RLK (RECEPTOR-like protein kinase) 25 | 1.23 |
| **PC-5p-2592253_1** | -10.88 | Cysteine-rich RLK (RECEPTOR-like protein kinase) 42 | 1.02 |
| **PC-5p-1977684_1** | -9.88 | Cysteine-rich RLK (RECEPTOR-like protein kinase) 26 | 1.12 |
| **PC-3p-719252_1** | -9.88 | Cysteine-rich RLK (RECEPTOR-like protein kinase) 26 | 1.00 |
| **PC-5p-1490392_1** | -9.88 | Cysteine-rich RLK (RECEPTOR-like protein kinase) 29 | 1.13 |
| **PC-5p-1501811_1** | -9.88 | Cysteine-rich RLK (RECEPTOR-like protein kinase) 34 | 1.81 |
| **PC-3p-2480743_1** | 0.18 | Cysteine-rich RLK (RECEPTOR-like protein kinase) 42 | 8.64 |
| **miR6196-p3** | 0.18 | Cysteine-rich RLK (RECEPTOR-like protein kinase) 14 | 1.09 |
| **miR5027-p3** | 2.86 | Cysteine-rich RLK (RECEPTOR-like protein kinase) 3 | 1.01 |
|  |  | TIR-NBS-LR |  |
| **miR472a** | -0.17 | Disease resistance protein (TIR-NBS-LRR class) | -1.00 |
| **miR393b-p5** | -9.88 | Disease resistance protein (TIR-NBS-LRR class) | -0.94 |
| **miR393b-p5** | -9.88 | Disease resistance protein (TIR-NBS-LRR class) | -0.80 |
| **miR845-p3** | -10.88 | Disease resistance protein (TIR-NBS-LRR class) | -0.30 |
| **miR845-p3** | -10.88 | Disease resistance protein (TIR-NBS-LRR class) | -0.26 |
| **miR169j-p5** | -9.88 | Disease resistance protein (TIR-NBS-LRR class) | -0.26 |
| **miR399k-p5** | -9.88 | Disease resistance protein (TIR-NBS-LRR class) | -0.25 |
| **miR169j-p5** | -9.88 | Disease resistance protein (TIR-NBS-LRR class) | -0.22 |
| **miR169j-p5** | -9.88 | Disease resistance protein (TIR-NBS-LRR class) | -0.20 |
| **miR414-p5** | -1.40 | Disease resistance protein (TIR-NBS-LRR class) | -0.19 |
| **miR169j-p5** | -9.88 | Disease resistance protein (TIR-NBS-LRR class) | -0.17 |
| **miR414-p5** | -1.40 | Disease resistance protein (TIR-NBS-LRR class) | -0.12 |
| **miR482a_2s** | 1.18 | Disease resistance protein (TIR-NBS-LRR class) | -0.09 |
| **miR169j-p5** | -9.88 | Disease resistance protein (TIR-NBS-LRR class) | -0.07 |
| **miR414-p5** | -1.40 | Disease resistance protein (TIR-NBS-LRR class) | -0.06 |
| **miR482a_2s** | 1.18 | Disease resistance protein (TIR-NBS-LRR class) | -0.05 |
| **miR2864-p5** | 10.06 | Disease resistance protein (TIR-NBS-LRR class) | -0.05 |
| **miR169j-p5** | -9.88 | Disease resistance protein (TIR-NBS-LRR class) | -0.03 |
| **miR169j-p5** | -9.88 | Disease resistance protein (TIR-NBS-LRR class) | -0.02 |
| **miR169j-p5** | -9.88 | Disease resistance protein (TIR-NBS-LRR class) | 0.06 |
| **miR169j-p5** | -9.88 | Disease resistance protein (TIR-NBS-LRR class) | 0.06 |
| **miR169j-p5** | -9.88 | Disease resistance protein (TIR-NBS-LRR class) | 0.08 |
| **miR472b** | -0.22 | Disease resistance protein (TIR-NBS-LRR class) | 0.30 |
| **miR472b** | -0.22 | Disease resistance protein (TIR-NBS-LRR class) | 0.37 |
| **miR472b** | -0.22 | Disease resistance protein (TIR-NBS-LRR class) | 0.50 |
|  |  | CC-NBS-LRR |  |
| **PC-5p-1349045_1** | -9.88 | Disease resistance protein (CC-NBS-LRR class) family | -0.08 |
| **miR482b** | 0.02 | Disease resistance protein (CC-NBS-LRR class) family | -0.30 |
| **miR482b** | 0.02 | Disease resistance protein (CC-NBS-LRR class) family | 0.21 |
| **miR398a-p5** | 10.06 | Disease resistance protein (CC-NBS-LRR class) family | -0.65 |
|  |  | Pathogenesis-Related |  |
| **MIR1509b-p3** | -0.99 | Pathogenesis-related thaumatin superfamily protein | 0.14 |
| **MIR408-p3** | 0.77 | Pathogenesis-related thaumatin superfamily protein | 0.10 |
| **MIR5517-p5** | 1.18 | pathogenesis-related 4 | -1.02 |
| **MIR3447-p3** | 11.06 | pathogenesis related homeodomain protein A | -0.28 |
|  |  |  |  |
| **MIR3434-p3** | 11.64 | EDS1 | -0.07 |
| **MIR4248b-p3** | -1.23 | PAD4 | -0.15 |
| **MIR4248b-p3** | -1.23 | EDS5 | 0.64 |
| **PC-5p-1150271_1** | 10.06 | SID2 | -0.19 |
|  |  |  |  |
| **PC-5p-1312398_1** | -10.88 | OPR3 | 0.92 |
|  |  |  |  |
| **MIR393b-p5_1** | -9.88 | JAR1 | 0.35 |
|  |  |  |  |
| **PC-3p-272434_3** | -1.82 | MPK4 | -0.20 |

Table S8 Primers used in the RT-qPCR

| **miR** | **Primer Sequences** | **Target gene annomination** | **Primer Sequences** |
| --- | --- | --- | --- |
| **miR172g-3p** | **GGGAATCTTGATGATGCTG** | AP2/ERF superfamily transcription factor | **TACCAACACAAGCGCCATTA** |
|  |  |  | **AGCTGATGCTACAGCCAATC** |
| **miR171i-p3** | **TTGAGCCGTGCCAATATC** | GRAS family transcription factor gene | **TCTTCTTCTCAAGGCGGAGG** |
|  |  |  | **GCTCTTGACTAGGAGAACCTG** |
| **miR393_R+2** | **TCCAAAGGGATCGCATTG** | F-box/RNI-like superfamily gene | **GGTTGGGGAGGTTGTGTTTA** |
|  |  |  | **AACCCTCACAAGACGAAAGC** |
| **miR828a** | **CTTGCTCAAATGAGTATT** | MYB transcription factor gene | **CTTGGCAACAGGTGGTCTTT** |
|  |  |  | **TCCTGGAATTCGCCCCTAA** |
| **miR172b-5p** | **GGAGCATCATCAAGATTCAC** | WRKY70 gene | **TCTGGTGTGCCTAAGTTTAAGT** |
|  |  |  | **GGAAACGTTCCAGTTGTTGG** |
| **miR482a** | **TTTCCAATTCCACCCATTCC** | TIR-NBS-LRR gene | **GACTATGCTTCTTCGAGGTGG** |
|  |  |  | **CAACAAATGCTGTGGCGAAG** |
| **miR5032-p3** | **CGTCCCTATGAAATGAGGC** | GroES-like zinc-binding dehydrogenase family protein gene | **TGTGACTGAGGTTCAACCAG** |
|  |  |  | **TCATCATGACTCCAACACCAG** |
| **miR6485-p3** | **TATCCACTCATGTAGTA** | O-methyltransferases gene | **ACAAGAACAAACCTTACCTTGTC** |
|  |  |  | **ACGTGATTGGTCAAGGTTGC** |
| **miR171e-p5** | **TGTTGGCAGAGCTCAATC** | epsin N-terminal homology (ENTH) domain-containing protein gene | **CGTGGCGTTGAAAACATTGA** |
|  |  |  | **AACTCGAATGCGCTTCATCT** |
| **miR6468** | **GGAGTGATTCAGGGAACC** | HXXXD-type acyl-transferase family gene | **GATTTCCTCCATCTGACTGCC** |
|  |  |  | **TGTTAGGCTGCCGACGATAG** |
| **5.8S ribosomal RNA*** | **GTCTGCCTGGGTGTCACGCAA** |  |  |
|  |  |  |  |

*Reference : Lu S, Sun YH, Chiang VL (2008) Stress-responsive microRNAs in *Populus*. Plant J 55(1):131-151.
